# Recent Developments in Nanoparticle‐Hydrogel Hybrid Materials for Controlled Release

**DOI:** 10.1002/advs.202507209

**Published:** 2025-07-30

**Authors:** Yiping Fan, Qi Han, Haiyan Li, Xudong Cai, Brendan Dyett, Ruirui Qiao, Calum J. Drummond, San H. Thang, Jiali Zhai

**Affiliations:** ^1^ School of Science, STEM College RMIT University Melbourne VIC 3000 Australia; ^2^ School of Engineering, STEM College RMIT University Melbourne VIC 3000 Australia; ^3^ Australian Institute for Bioengineering and Nanotechnology The University of Queensland Brisbane QLD 4072 Australia; ^4^ School of Chemistry Monash University Clayton VIC 3800 Australia

**Keywords:** drug delivery, hydrogel, hydrogel composite, hydrogel hybrid, lipid, microgel, nanoparticles, organic–inorganic hybrid, stimuli responsiveness

## Abstract

Nanoparticle (NP)–hydrogel hybrid materials have emerged as promising platforms for controlled drug delivery, combining the tunable chemistry of NPs (e.g., liposomes, polymeric, and inorganic NPs) with the porous, biocompatible networks of hydrogels (e.g., alginate or poly(ethylene glycol)‐based systems). These composites can encapsulate a wide range of bioactive agents—small molecules, peptides, proteins, and nucleic acids—within hydrogel matrices, guided by molecular interactions such as electrostatic forces, hydrogen bonding, and hydrophobic/hydrophilic balance. Such interactions influence both the physicochemical stability and drug release profiles of the system. This review highlights recent advances in NP–hydrogel composites, emphasizing how molecular‐level interactions shape the nanostructure, drug encapsulation, and release behavior. The enhanced mechanical strength, stimuli responsiveness, pharmacokinetics, and biological performance of these materials are also discussed. Particular focus is placed on how improved mechanistic understanding can guide the design of next‐generation hybrid systems with tunable, predictable release for biomedical applications. This review provides a comprehensive overview of NP–hydrogel hybrid materials as versatile drug delivery systems and outlines future research directions for their use in personalized therapy, targeted treatment, and broader clinical translation.

## Graphical abstract

This review highlights recent advances in nanoparticle–hydrogel hybrid materials for controlled drug delivery. It explores diverse nanocarrier–hydrogel combinations, drug loading strategies, release mechanisms, and stimuli‐responsive behaviors. Emphasis is placed on the molecular interactions driving hybrid material performance, offering insights into rational design for applications in cancer therapy, wound healing, antimicrobial delivery, and gene‐based therapeutics

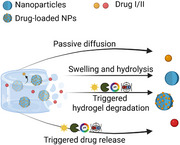

## 1 Introduction

Advances in smart materials‐based drug delivery have seen tremendous growth over the past few decades.^[^ ^1^, ^2^, ^3^, ^4^, ^5^ ^]^ Nanoparticle (NP)‐based drug delivery carriers including lipid NPs (LNPs),^[^ ^6^, ^7^, ^8^, ^9^, ^10^, ^11^ ^]^ polymeric NPs,^[^ ^7^, ^12^, ^13^, ^14^ ^]^ silica,^[^ ^6^, ^15^, ^16^ ^]^ and carbon‐based^[^ ^9^, ^17^, ^18^, ^19^ ^]^ NPs, and inorganic NPs, such as silver (Ag), gold (Au), titanium, metal oxide, and metal alloy^[^ ^20^, ^21^, ^22^, ^23^ ^]^ have been developed as drug delivery systems for many biomedical applications, including cancer therapy,^[^ ^24^, ^25^, ^26^, ^27^ ^]^ gene therapy,^[^ ^28^, ^29^ ^]^ and antimicrobial treatment and control.^[^ ^30^, ^31^ ^]^ NP‐based drug delivery has several advantages including high drug loading, protection of drug from premature release, stimuli responsiveness, biological barrier crossing, and cell and/or organ targeting afforded by tunable particle size, structure, stiffness, surface charge, and surface modification.^[^ ^32^, ^33^ ^]^ Furthermore, NP delivery systems can enable controlled release of encapsulated drugs responding to either internal or external stimulus^[^ ^34^ ^]^ through changes in pH,^[^ ^26^, ^35^, ^36^, ^37^ ^]^ temperature,^[^ ^38^ ^]^ magnetic field,^[^ ^39^, ^40^ ^]^ light,^[^ ^40^, ^41^ ^]^ oxidation‐reduction (redox) reactions,^[^ ^26^, ^42^ ^]^ and enzyme environment. However, for clinical translation, NP drug delivery systems still need to consider potential issues, such as low stability, fast clearance in bloodstream, low bioavailability, liver tropism, limited ability to cross biological barriers, and off‐target systemic distribution.^[^ ^43^, ^44^, ^45^, ^46^ ^]^ These challenges can arise due to the formation of protein coronas, opsonization, and phagocytosis in the bloodstream which could immediately alter the NP's physicochemical and targeting properties.^[^ ^47^, ^48^, ^49^, ^50^ ^]^

In recent years, integrating NPs into hydrogels has provided a pathway to address many of the aforementioned potential challenges in drug delivery while combining the unique properties of the hydrogel bulk system to broaden the application into tissue engineering and wound healing.^[^ ^51^, ^52^, ^53^, ^54^, ^55^ ^]^ For example, a hydrogel coating over LNPs protected drug loaded NPs from the acidic environment of the stomach and enabled on‐target drug delivery for colon cancer therapy.^[^ ^56^ ^]^ In addition, the drug encapsulation of hydrophobic drugs in hydrogel systems can be improved by preloading into amphiphilic polymer and LNPs.^[^ ^57^, ^58^, ^59^ ^]^ Drug release from the hybrid materials can be accelerated by applying external stimuli, such as through a magnetic field on hybrid materials with magnetic NP embedment.^[^ ^60^ ^]^ On the other hand, incorporation of NPs introduces multifunctionalities to hybrid materials, such as the hyperthermia effect of magnetic NPs^[^ ^61^, ^62^ ^]^ and the unique properties of NPs promote tissue penetration and on‐target drug delivery^[^ ^63^ ^]^ thus improve the therapeutic efficacy and outcomes. Overall, NP‐hydrogel hybrid materials provide outstanding advantages over a single NP or hydrogel system in controlled drug delivery.

A hydrogel is a 3D, cross‐linked polymer network^[^ ^64^, ^65^, ^66^ ^]^ and has been widely used in biomedicine, tissue engineering, wound healing, and 3D bioprinting due to unique chemical and physical properties, mechanical strength, high biocompatibility, mimicry of extracellular matrix, porosity, injectability, and support to cell growth.^[^ ^67^, ^68^, ^69^ ^]^ However, due to the very high water content of hydrogels, delivery of hydrophobic drugs and controlled release are challenges for hydrogel systems which generally have low drug loading capacities and poor controlled release. Additionally, for hydrophilic drugs, leakage and burst release are usually observed after in vivo application. In addition to the traditional bulk hydrogel, microgels, and nanogels are micro‐ and nanosized particles containing the cross‐linked polymer networks which have recently gained attention for drug delivery systems to exploit their particle properties.^[^ ^70^, ^71^, ^72^, ^73^ ^]^

**Figure 1:**
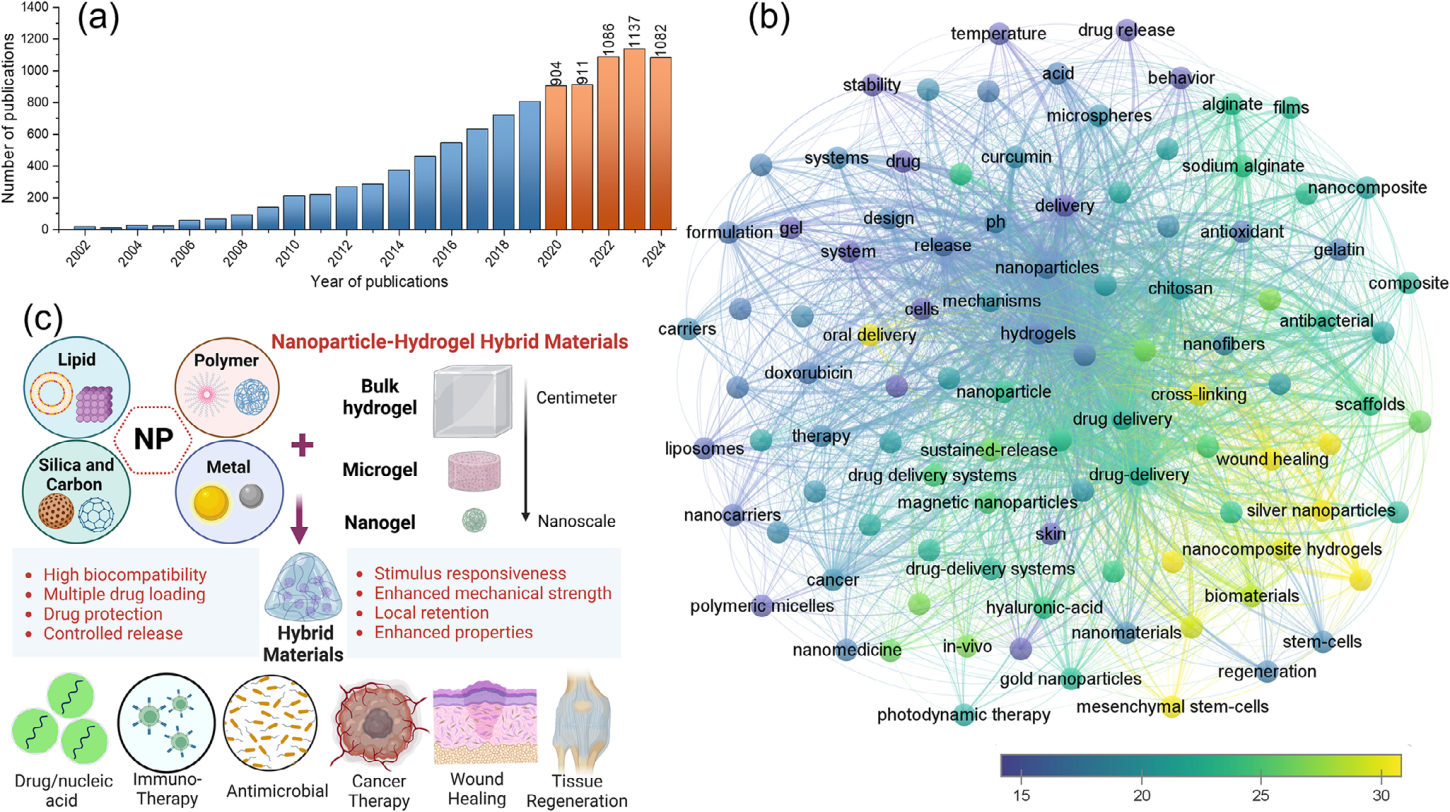
a) Number of publications (2000–2024, recent 5 years highlighted in orange) retrieved from Web of Science using keywords “(hydrogel*) AND (nanoparticle* OR nanomaterial*) AND (“controlled release” OR “drug delivery”) AND (drug* OR “small molecule*” OR chemotherapeutic* OR antimicrobial* OR “protein drug*” OR peptide* OR “nucleic acid*” OR biomolecule* OR “mRNA*”). b) Keyword co‐occurrence analysis (5120 papers, 2020–2024) visualized using VOSviewer, Nodes with color gradients (blue to yellow) indicating low to high average citation frequencies, Key terms, such as “nanoparticles,” “hydrogels,” and “drug delivery” dominate, emphasizing their research prominence. c) Design and biomedical applications of NP‐hydrogel hybrid materials and their advantages.

The emergence of NP‐hydrogel hybrid materials aims to overcome the potential drawbacks of utilizing NPs or hydrogels alone as drug delivery systems. Research in NP‐hydrogel hybrid materials has seen a remarkable growth over the past two decades in various disciplines,^[^ ^67^, ^74^, ^75^, ^76^ ^]^ with the number of publications increasing nearly 1000‐fold according to a Web of Science database search using keywords of “nanoparticle” and “hydrogel” and “drug delivery” (**Figure** **1**a), The co‐occurrence analysis of over 5000 publications in 2020–2024, by correlating the most important 100 terms identify core research themes around “nanoparticles,” “hydrogels,” and “drug delivery,” with terms like “cancer therapy,” “wound healing,” “antimicrobial,” and “tissue regeneration,” highlights the versatility and multifunctionality of these materials (Figure 1b). In addition, high citation frequencies associated with “drug delivery” and “biomedical applications” suggest strong research and clinical relevance. Highly cited terms, such as “chitosan,” and “sodium alginate” highlight the increasing interest on improving biocompatibility with natural polymers. Peripheral terms, including “emulsion‐based hydrogels” and “solid lipid nanoparticles,” suggest diverse fabrication methods, while links to “mRNA” and “protein drug delivery” indicate a growing focus on delivery of biologics and nucleic acids. This reflects increasing interest in combining the advantages of hydrogel biocompatibility, water retention, and biological structure mimicry with the unique properties of high drug loading and controlled delivery of colloidal NPs. To date, almost all main types of NPs from inorganic to organic materials have been embedded into polymeric hydrogel matrices to create novel hybrid materials with superior properties and tailored functionalities for cancer therapy, wound healing, drug delivery, inter alia^[^ ^77^, ^78^ ^]^ (Figure 1c). Zhong et al., reviewed and summarized the delivery of RNA via NP‐hydrogel hybrid materials, focusing on the molecular interactions between RNA and different types of nanocarriers, controlled release, and related biomedical applications.^[^ ^74^ ^]^ Ahmad et al., highlighted the growing need for antimicrobial strategies due to the limitations of current treatments and the rise of antibiotic resistance and also explored NP‐hydrogel hybrid systems as promising biomaterials for controlled, sustained, and targeted antimicrobial agent delivery.^[^ ^79^ ^]^ Choi et al., also reviewed briefly the approach of creating NP‐hydrogel hybrid systems and highlighted their advantages for drug delivery including enhanced loading, stimuli responsiveness, enhanced mechanical strength, and prevention of aggregation.^[^ ^67^ ^]^

Previous literature reviews had less emphasis on the interactions inside the drug‐NP‐hydrogel networks, occurring during the fabrication stage as well as the passive or stimuli‐induced release stage, yet these interactions control the critical physicochemical properties and release mechanism of the formed drug delivery system. In addition, these interactions may change the hydrogel properties, such as porosity, swelling, viscoelasticity, mechanical strength, and biocompatibility. This review aims to use the most recent developments in the NP‐hydrogel hybrid materials field to explore the molecular interactions employed for the modular design of embedding drug‐loaded NPs into polymeric hydrogel networks, and phenomena during the subsequent drug release mechanism from such systems. Using specific examples, key properties of hybrid systems are also highlighted to emphasize considerations which are important for various drug delivery vehicle design purposes. Current gaps in knowledge are identified to guide future studies in the area. By better understanding the governing forces and mechanisms in drug‐NP‐hydrogel systems, future design of hybrid systems can be more efficient leading to solutions to current problems in drug delivery.

## 2 NP‐Hydrogel Hybrid Material Design

### 2.1 Overview of NP‐Hydrogel Hybrid Materials

**Figure 2:**
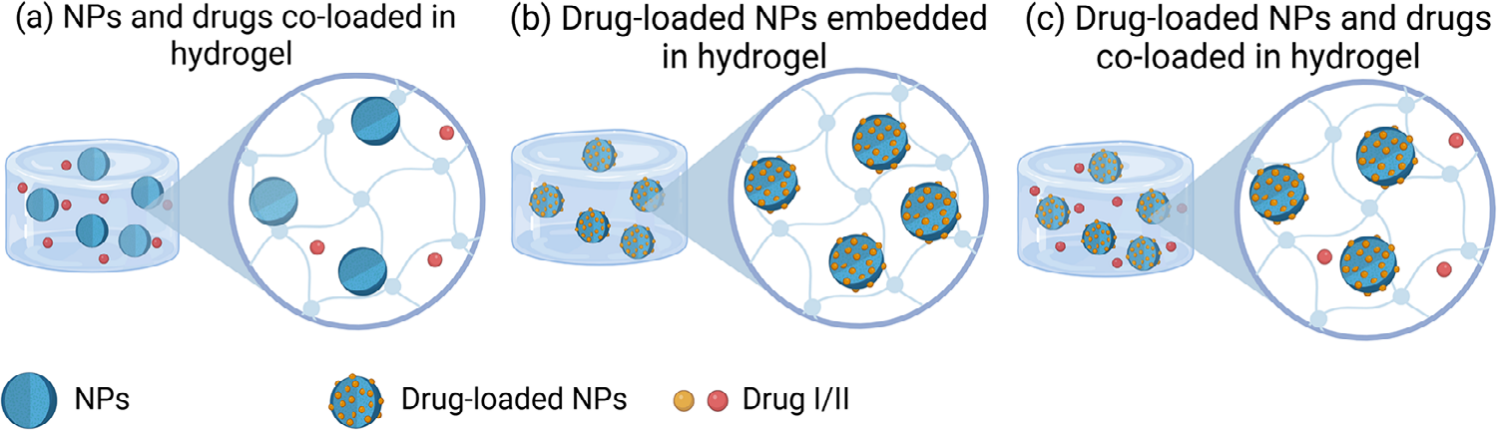
Illustration of different drug‐NP‐hydrogel hybrid materials. a) Loading NPs and free drugs in hydrogel networks. b) Loading drug‐loaded NPs in hydrogel networks. c) Loading drug‐loaded NPs and a secondary free drug in hydrogel networks.

The development of high‐performance NP‐hydrogel hybrid materials holds the promise of leveraging the advantages of both hydrogel and NP drug delivery systems, such as enhanced biocompatibility, controlled drug release, reduced systemic clearance, enhanced local retention, and stimuli‐responsive delivery to specific targets. To date, many types of NPs, including polymeric NPs,^[^ ^80^, ^81^, ^82^ ^]^ LNPs,^[^ ^83^, ^84^, ^85^ ^]^ carbon‐ and silica‐based NPs,^[^ ^86^, ^87^, ^88^ ^]^ and metal NPs,^[^ ^89^, ^90^, ^91^ ^]^ have been incorporated into hydrogel materials made from either natural or synthetic polymers. The NP‐hydrogel composite system mainly exists in three multicomponent architectures: free bioactive agents and NPs coloaded into the hydrogel network (**Figure** **2**a),^[^ ^60^, ^92^, ^93^, ^94^ ^]^ preloading drugs or bioactive compounds into NPs then incorporating into the hydrogel network (Figure 2b),^[^ ^80^, ^82^, ^83^, ^87^ ^]^ or integrating bioactive agents‐loaded NPs with a secondary drug into the hydrogel network^[^ ^58^ ^]^ (Figure 2c).

It should be noted that when free drug and NPs are separately coloaded into hydrogels. NPs are often added to the hybrid material as an entity that introduces extra functionality to facilitate therapeutic treatment or enhance the mechanical and other properties of the hydrogel (Figure 2a). For example, iron oxide NPs and 2D nanosheets (MXenes) have been incorporated into hydrogels for their inherent property to respond to a near infrared (NIR)‐triggered photothermal effect, providing effective treatments for wound dressing and cancer therapy.^[^ ^92^, ^93^, ^94^ ^]^ Moreover, the release of doxorubicin (DOX), an anticancer drug, is accelerated by the magnetic field triggered hydrogel swelling when embedding iron oxide NPs into chitosan hydrogel.^[^ ^60^ ^]^ For drug loading in NPs and then embedded in hydrogel (Figure 2b). NPs usually serve as a nanocarrier that provides protection to fragile bioactive agents, such as mRNA,^[^ ^29^ ^]^ or increases the loading capacity of hydrophobic drugs, such as cisplatin,^[^ ^56^ ^]^ DOX,^[^ ^95^ ^]^ and paclitaxel (PTX).^[^ ^96^ ^]^ For loading drug‐loaded NPs together with a secondary drug in hydrogel (Figure 2c), different drugs offer synergistic effects and can be released with different rates in a controlled manner, and the NPs can improve the targeted delivery of encapsulated drug.^[^ ^58^ ^]^ For example, oxaliplatin and RNA‐loaded LNPs are embedded in a hyaluronic acid (HA) and alginate‐based hydrogel for mediating triple immune effects against postsurgery colorectal cancer recurrence,^[^ ^58^ ^]^ where oxaliplatin is released faster to kill the colorectal cancer cells first, and then the RNA‐loaded LNPs sequentially release the RNA for long‐term tumor suppression.

**Table** **1** provides an overview of widely used NPs in the field, highlighting their physical properties, material types, and morphologies, as the selection of NPs, either for higher drug loading or for improving other properties of the hydrogel system, is a critical consideration for fabricating the hybrid materials. The physicochemical properties and the drug loading ability and controlled release by the selected NPs is central to the design of the hybrid materials. For instance, spherical NPs often enable uniform drug loading and controlled release, while rod‐shaped or sheet‐like silica based and metal‐based NPs reinforce the structure of hybrid materials.^[^ ^93^, ^97^, ^98^ ^]^ Moreover, due to the amphiphilic properties of self‐assembled polymeric and LNPs, it enables the efficient encapsulation and high drug loading of hydrophobic drugs^[^ ^3^, ^99^ ^]^ and fragile nucleic acid agents, such as mRNAs.^[^ ^29^ ^]^ Table 1 also includes “Drug Loading/Release” properties, offering insights into their potential for sustained release, pH‐sensitive release, or targeted drug delivery.

**Table 1:** Commonly used NPs in hybrid materials and their properties.

NP type^a)^	Size range	Porosity	Material type	Shape and morphologies	Drug loading/release	Refs.
Polymeric NPs	Large: 10 nm to 1 µm	Porous or nonporous	Biodegradable polymers (e.g., PLGA, PEG)	Spherical, cubic, or irregular	Sustained release, biodegradable, nontoxic	[13]
‐ Dendrimers	Large: 1–100 nm	Nonporous	Synthetic polymers (e.g., PAMAM)	Highly branched, often spherical	High drug loading capacity, targeted delivery, functional versatility	[100]
‐ Pluronic F127	10–100 nm	Nonporous	Polymeric and mostly hybrid with others	Spherical as micelles	Targeted drug delivery, improves cell membrane interaction	[101]
‐ Tannic Acid	50–300 nm	Nonporous	Tannic acid with natural polymer functionality	Spherical	For hydrophobic drug loading, controlled release	[102]
‐ PLGA‐PEG	50–500 nm	Nonporous	Polymer (PLGA) with PEG coating	Irregular	Biodegradable, encapsulation for sustained release	[95]
Natural polymeric NP: e.g., Alginate, Chitosan	Large: 50–500 nm	Nonporous	Natural (e.g., alginate, chitosan)	Irregular or spherical	Biocompatible, suitable for controlled release, enhances bio‐adhesion	[103, 104, 105]
Lipid NPs	Large: 50 nm to 1 µm	Porous	Lipid	Spherical, cubic, or irregular	Effective for hydrophobic drug and nucleic acid loading, pH‐sensitive release	[10]
‐ Liposomes	Large: 50 nm to 1 µm	Porous	Lipid bilayer	Spherical	For both hydrophilic and hydrophobic drugs, and large bioactives, enhances bioavailability	[11]
‐ Cubosomes	Large: 100–500 nm	Porous	Lipid bilayer (bicontinuous cubic phase)	Cubic internal structure		[11]
Silica and carbon‐based NPs	Small: 20–200 nm	Mesoporous or nonporous	Silica or carbon derivatives	Spherical, rods, tubular	Suitable for controlled release, surface functionalization for targeting	[106, 107]
‐ MSNs	50–200 nm	Mesoporous	Silica	Spherical	High surface area for drug loading, sustained release	[108]
‐ Nano Silicate	≈100 nm	Nonporous	Silicate‐based materials	Irregular	Can be modified for drug encapsulation and controlled release	[97]
‐ Carbon	1–100 nm	Nonporous	Carbon	Tubular	Drug delivery via functionalization, nontoxic	[18]
Metal‐Based NPs	Small: 1–100 nm	Nonporous	Metals (e.g., Au, Ag) or metal oxides	Spherical, rods, cubic, or sheets	Targeted drug delivery, imaging, therapy, enhances hydrogel properties for antimicrobial applications	[109]
‐ Gold NPs	1–50 nm	Nonporous	Noble metal (Au)	Spherical, rods	Drug delivery with targeted therapy, surface functionalization for drug attachment, photothermal applications	[110]
‐ Silver NPs	1–100 nm	Nonporous	Noble metal (Ag)	Spherical, rods	Antimicrobial treatment, enhances mechanical, and antibacterial properties of hydrogels	[111]
‐ MXene	≈50 nm (lateral size)	Nonporous	2D carbide material	Sheet‐like	Targeted drug delivery, can be modified for specific release profiles, structural reinforcement	[112, 113]
‐ Iron Oxide NPs	10–100 nm	Nonporous	Magnetic iron oxide	Spherical	Magnetic targeting, controlled release via magnetic fields, useful in hyperthermia therapy	[114]

^a)^ Abbreviations: Poly(lactic‐*co*‐glycolic) acid (PLGA), Polyethylene glycol (PEG), Poly (amidoamine) (PAMAM), Mesoporous silica NPs (MSNs).

On the other hand, a hydrogel network can provide a 3D, water‐rich matrix that mimics biological tissues, enabling biocompatibility, sustained release, and support for cell and tissue growth.^[^ ^115^ ^]^ Chitosan,^[^ ^60^, ^111^, ^116^, ^117^ ^]^ HA,^[^ ^98^, ^111^, ^118^ ^]^ and sodium alginate^[^ ^58^, ^114^, ^119^, ^120^ ^]^ are commonly used natural polymers in NP‐hydrogel hybrid materials. These polymers are generated from natural products, and can make the hybrid materials highly biocompatible, and suitable for regenerative medicine and drug delivery. Agarose^[^ ^95^, ^112^, ^113^ ^]^ is another commonly used natural polymer with a unique temperature dependent sol–gel phase change, When combined with thermal responsiveness of metal‐based NPs, it introduces control over drug release to the NP‐hydrogel hybrid materials. Moreover, synthetic polymers, such as polyethylene glycol (PEG)^[^ ^102^, ^121^, ^122^, ^123^ ^]^ and poly(lactic‐*co*‐glycolic acid) (PLGA),^[^ ^95^, ^98^ ^]^ show tunable properties and stabilities which can be easily adjusted to optimize mechanical and physical properties for specific applications.

**Table** **2** summarizes recent developments (since 2020) of the drug‐NP‐hydrogel hybrid materials, highlighting the type of NPs, hydrogel materials, loaded bioactives, the mechanism of drug loading and controlled release, and biomedical applications. There is an extensive material library of drugs, NPs and polymers to explore, and multiple drugs. NPs and polymers can be combined in one single hybrid drug delivery system. For instance, LNPs and polymeric NPs‐hydrogel hybrid materials are widely studied in immunotherapy and cancer therapy, while carbon‐, silica‐, and metal‐based NPs‐hydrogel hybrid materials are used with enhanced mechanical properties in tissue regeneration. Moreover, metal‐based NPs, are also used in tumor suppression and antimicrobial applications. In addition, there are other types of NPs including virus‐like biological NPs, protein NPs, bio‐mimicking NPs, black phosphorus NPs, and bio‐clay NPs, which show unique properties in biomedical applications.

**Table 2:** Recently developed NP‐hydrogel hybrid nanomaterials published since 2020 and their properties and applications.

NP materials^a)^	Hydrogel materials	Drugs	Embedding mechanism	Release mechanism	Application	Refs.
Polymeric NPs						
Polymeric – Pluronic F127 micelles	PVP microneedles	Imiquimod (R837)	Hydrophobic interaction, NP‐ Physical embedment.	Hydrogel degradation (hydrolysis).	Cancer immunotherapy.	[101]
Natural Polymeric – Janus‐structured dextran/chitosan‐bismuth selenide	Calcium gluconate	Bismuth selenide nanosheet; Janus NP as antibacterial agent	Chemical, NP‐ Physical embedment.	Adhesion to biofilm and NIR light trigger.	Photothermal treatment for bacterial killing, Anti‐ drug‐resistant bacterial, Biofilm eradication, Wound dressing.	[124]
Polymeric – Tannic acid	CMC and PEG based hydrogel	Antagomir‐21, a cholesterol‐modified miRNA inhibitor	Hydrophobic interaction, NP‐ Physical embedment.	ROS and pH.	Gene therapy.	[102]
Polymeric – CpG self‐crosslinked NP with PEI and genipin	IR820 conjugated α‐CD with PEG	CpG	Self‐crosslinked, NP‐ Physical embedment.	NIR.	Tumor treatment. Synergistic immunotherapy and photothermal therapy.	[121]
Polymeric – PEI	PEG‐4000 with α‐ CD	DOX and CpG	DOX‐ chemical (carbodiimide crosslinking reaction), CpG‐ electrostatic interaction. NP‐ Physical embedment, co‐loaded with dendritic cells.	NIR and pH.	Cancer therapy. Synergistic immunotherapy and photothermal therapy	[122]
Polymeric – PLGA	HA	Transforming growth factor‐β inhibitor	Hydrophobic interaction, NP‐ Physical embedment.	Hydrogel degradation (passive).	Scarless skin wound healing.	[98]
Polymeric – PLGA‐PEG	Agarose	Imiquimod (R837) and DOX	R837‐ Hydrophobic interaction, DOX‐ physical embedment. NP‐ physical embedment.	Hydrogel softening (photothermal with NIR).	Photothermal and immunotherapy for breast tumor.	[95]
Polymeric – TPT	HA	Lonidamine and DOX	Lonidamine‐Chemical (sulfur bond) DOX‐ hydrophobic interaction and π–π stacking, NP‐ physical embedment.	Lonidamine‐ redox. Hydrogel degradation (enzyme)	Mitochondria targeting. Chemo‐ and immune‐combinational therapy.	[57]
Polymeric – mPEG_45_‐PASP(DIP)_60_‐PPHE_30_ triblock copolymer	Caffeic acid‐grafted ε‐polylysine and phenylboronic acid‐grafted oxidized dextran.	Mangiferin and diclofenac sodium	Mangiferin‐ hydrophobic interaction, Diclofenac sodium‐ physical embedment, NP‐ physical embedment.	ROS and pH.	Wound healing.	[125]
^Lipid NPs^						
Lipid – DOTAP with cancer cell membrane	Alginate and HA	A short hair‐pinned RNA. Oxaliplatin	RNA‐ Hydrophobic interaction, Oxa‐ Physical embedment, NP‐ Physical embedment,	Passive diffusion and hydrogel degradation (hydrolysis).	Immunotherapy.Suppressing postsurgical tumor relapse.	[58]
Lipid – Trilaurin	Dextran	Cisplatin/superparamagnetic iron oxide NPs	Hydrophobic interaction, NP‐ Physical embedment.	Hydrogel degradation (enzyme).	Colon cancer thermotherapy and chemotherapy.	[56]
Lipid – DOTAP with cancer cell membrane	Alginate and HA	Cancer cell membrane with T‐cell immunoglobulin mucin blockade nano‐vaccine	Hydrophobic interaction, NP‐ Physical embedment.	Passive diffusion and hydrogel degradation (hydrolysis).	Immunotherapy.Suppressing postsurgical tumor relapse	[119]
Lipid – Phytantriol cubosomes	HA	Diclofenac	Hydrophobic interaction, NP‐ Physical embedment.	Hydrogel degradation (hydrolysis).	Antiosteoarthritis and anti‐inflammatory.	[118]
^Carbon and Silica based NPs^						
Diatomite biosilica	PDA and CMC	Budesonide	Hydrophobic interaction, NP‐ physical embedment.	Hydrogel degradation (pH).	Allergic rhinitis treatment. Antibacterial and anti‐inflammation.	[126]
Nano silicate	GelMA	Metformin	Passive absorption, NP‐ electrostatic interaction.	Passive diffusion.	Wound dressing.	[97]
MSNs	HA	DOX and GCN5 siRNA	DOX‐ Passive absorption, siRNA‐ electrostatic interaction NP – electrostatic interaction,	pH and redox.	Cancer immunotherapy for tumors with multidrug resistance.	[108]
Silica	Sodium alginate	Rosuvastatin	Passive absorption, NP‐ physical embedment	Passive diffusion.	Bone tissue regeneration.	[120]
Silica	Chitosan	Ciprofloxacin	Passive absorption, NP‐ physical embedment.	pH.	Bone tissue regeneration.	[116]
MSNs	PEG and HA	Silver	Chemical (reduction), NP‐ covalently embedded.	pH.	Antibacterial. Wound healing.	[127]
MSNs	PEG ‐poly(b‐aminoester urethane)	CPT	Passive adsorption, NP‐ electrostatic interaction.	pH and temperature.	Immunotherapy. Implantable delivery depot.	[123]
Porous silicon	Poloxamer 407	Mometasone	Solvent evaporation method, NP‐ physical embedment.	Passive diffusion.	Intranasal administration, Rhinosinusitis treatment.	[128]
^Metal–based NPs^						
Iron oxide	Alginate	DOX	Physical embedment, NP‐ physical embedment.	Hydrogel degradation (pH and redox). Targeting release with magnetic field.	Cancer therapy	[114]
Copper sulfide	HA	Deferoxamine	Physical embedment, NP‐ physical embedment.	NIR and hydrogel degradation (enzyme).	Photothermal therapy. Antibacterial. Diabetic pressure ulcers treatment. Wound healing.	[61]
Silver	Chitosan and HA	Silver NPs and Nystatin	Physical embedment, NP‐ physical embedment.	Passive diffusion.	Antimicrobial and antifungal. Wound healing.	[111]
Calcium peroxide‐indocyanine green combined with lauric acid and manganese dioxide	PDA and HA	Oxygen and singlet oxygen ^1^O_2_	Physical embedment, NP‐ physical embedment.	Reaction. Hydrogel degradation (NIR).	Photothermal therapy. Wound healing and anti‐inflammation.	[129]
Bimetallic oxides	Guggul gum grafted polyacrylamide hydrogel	Naproxen	Passive absorption, NP‐ polymerization with hydrogel.	Passive diffusion.	Wound healing, antidiabetic, radical scavenging, antibacterial, hemolytic activity. Antioxidant.	[130]
Zeolitic imidazolate framework‐8@ceric oxide	GelMA	DOX	Chemical (metal–organic framework), NP‐Physical embedment.	NP degradation (pH)	Anticancer, tumor recurrence prevention, ROS scavenging and wound healing.	[131]
MXene (Ti_3_C_2_)	Agarose	DOX	Physical embedment, NP‐ hydrogen bonding.	Hydrogel melting (NIR‐ induced thermal)	Photothermal therapy. Chemotherapy.	[112]
MXene (Ti_3_AlC_2_)	Agarose	DOX	Physical embedment, NP‐ hydrogen bonding.	Hydrogel melting (NIR‐ induced thermal)	Antitumor.	[113]
Niobium carbide	PDA	Imiquimod (R837)	Passive absorption, NP‐ physical embedment.	NP degradation (NIR‐II)	Photothermal therapy and immunotherapy.	[132]
Galinstan liquid metal and iron oxide	Alginate and HA	ROS	Hydrophobic interaction, NP‐ physical embedment.	Fenton reaction (GSH and microwave).	Immunotherapy, chemotherapy and hyperthermia treatment.	[133]
Iron oxide	Chitosan	DOX	Passive absorption, NP‐ physical embedment.	Magnetic field and hydrogel degradation (passive).	Chemotherapy and hyperthermia treatment.	[60]
Iron oxide	METAC	PTX	Hydrophobic interaction, NP‐free radical polymerization.	pH	Antitumor. Antibacterial.	[96]
Gold	α‐CD	Antigen peptide coloaded with CpG	Peptide‐surface adsorption (affinity), CpG – physically embedment, NPs – “host–guest” interaction.	Passive diffusion.	Cancer immunotherapy.	[110]
Titanium based	Self‐assembling peptide‐based hydrogel	Growth factor	Chemical (Thiol‐Maleimide crosslinking), NP‐Physical embedment.	UV‐responsive	Growth factor sustained delivery.	[134]
Au and Ag	Chitosan and acrylamide	Fluorouracil	Hydrogen bonding and Van der Waals interaction, Gold NPs‐chemical (reduction), Silver NP‐ chemical(reduction).	pH	Anticancer drug delivery. Antimicrobial.	[117]
^Other NPs^						
Black phosphorus quantum dots	NIPAM	Zoledronate	Passive absorption, NP‐ physical embedment.	Hydrogel degradation (NIR)	Breast cancer immunotherapy.	[135]
Virus‐like particles derived from the hepatitis B virus core	Dextran	CpG	Hydrophobic interaction, NP‐ Physical embedment.	Hydrogel degradation (enzyme)	Abscopal antitumor. Cancer immunotherapy.	[136]
Bioactive glass	Chitosan and silk fibroin	Copper and calcium ions	Stöber method, NP‐Physical embedment.	Passive diffusion and hydrogel degradation (passive).	Bone tissue regeneration.	[137]
Cellulose nanocrystals (CNCs) decorated with Fe_3_O_4_ nanoparticles	NIPAm	Vancomycin	Passive adsorption, NP‐ Physical embedment.	Hydrogel swelling (NIR)	Wound healing.	[138]

^a)^ Abbreviations: Cytosine phosphoguanosine (CpG), Polyvinylpyrrolidone (PVP), Hyaluronic acid (HA), Near infrared (NIR), Dioleoyl‐3‐trimethylammonium propane (DOTAP), Polyethylene glycol (PEG), Carboxymethyl chitosan (CMC), Reactive oxygen species (ROS), Polyethylenimine (PEI), Cyclodextrin (CD), Doxorubicin (DOX), Poly(lactic‐*co*‐glycolic) acid (PLGA), Polymer containing triphenylphosphine and poly(l‐lactic acid) (TPT), Poly(aspartic acid) (PASP), 2‐(Diisopropylamino)ethylamine (DIP), Poly(Phenyl ether) (PPHE), Polydopamine (PDA), Gelatin Methacryloyl (GelMA), Camptothecin (CPT), Glutathione (GSH), [2‐ (methacryloyloxy) ethyl] trimethyl ammonium chloride (METAC), Paclitaxel (PTX), *N*‐isopropylacrylamide (NIPAM).

According to the examples listed in Table 2, NP‐hydrogel hybrid materials show great advantage over a single drug delivery system of NPs or hydrogels alone. Incorporation of hydrogel can improve the biocompatibility, stability, and local retention, while incorporation of NPs can enhance the mechanical strength, functionality, drug loading capacity, and controlled drug release. For example, LNPs such as trilaurin‐based NPs are unstable under acidic environment, which may make it a less suitable nanocarrier for drug delivery with oral administration through the stomach. To that end, an acid‐durable hydrogel coating provides a promising solution, Lu et al., applied an acid‐durable dextran coating over trilaurin‐based LNPs, enhanced the overall hybrid material durability in the stomach, protected the system from LNP retention and premature drug release in stomach, and enabled a targeted on‐demand cisplatin drug delivery to colon for colon cancer therapy. The dextran layer was degraded by the specific colon enzyme and the drugs in the LNPs were only released to colon tumor.^[^ ^56^ ^]^ Moreover, embedding silver NPs in chitosan and HA^[^ ^111^ ^]^ and copper‐based NPs in HA^[^ ^61^ ^]^ controlled NP release and prevented NP leakage, thus improving biocompatibility while maintaining the antimicrobial effectiveness and photothermal behavior of the NPs, respectively. On the other hand, encapsulation of hydrophobic drugs in a hydrogel system is a significant challenge due to the high water content in hydrogels, while the amphophilic property of LNPs and polymeric NPs makes them an ideal nanocarrier to improve the drug loading efficiency. Researchers have reported successful encapsulation of hydrophobic anticancer bioactives in NP‐hydrogel composite materials with high drug loading efficiency, including DOX,^[^ ^57^, ^95^, ^122^ ^]^ cisplatin,^[^ ^56^ ^]^ Paclitaxel,^[^ ^96^ ^]^ DNA,^[^ ^121^ ^]^ RNA,^[^ ^58^, ^108^ ^]^ and peptides.^[^ ^98^, ^119^ ^]^

### 2.2 Drug Loading in NPs or in Hydrogels

**Figure 3:**
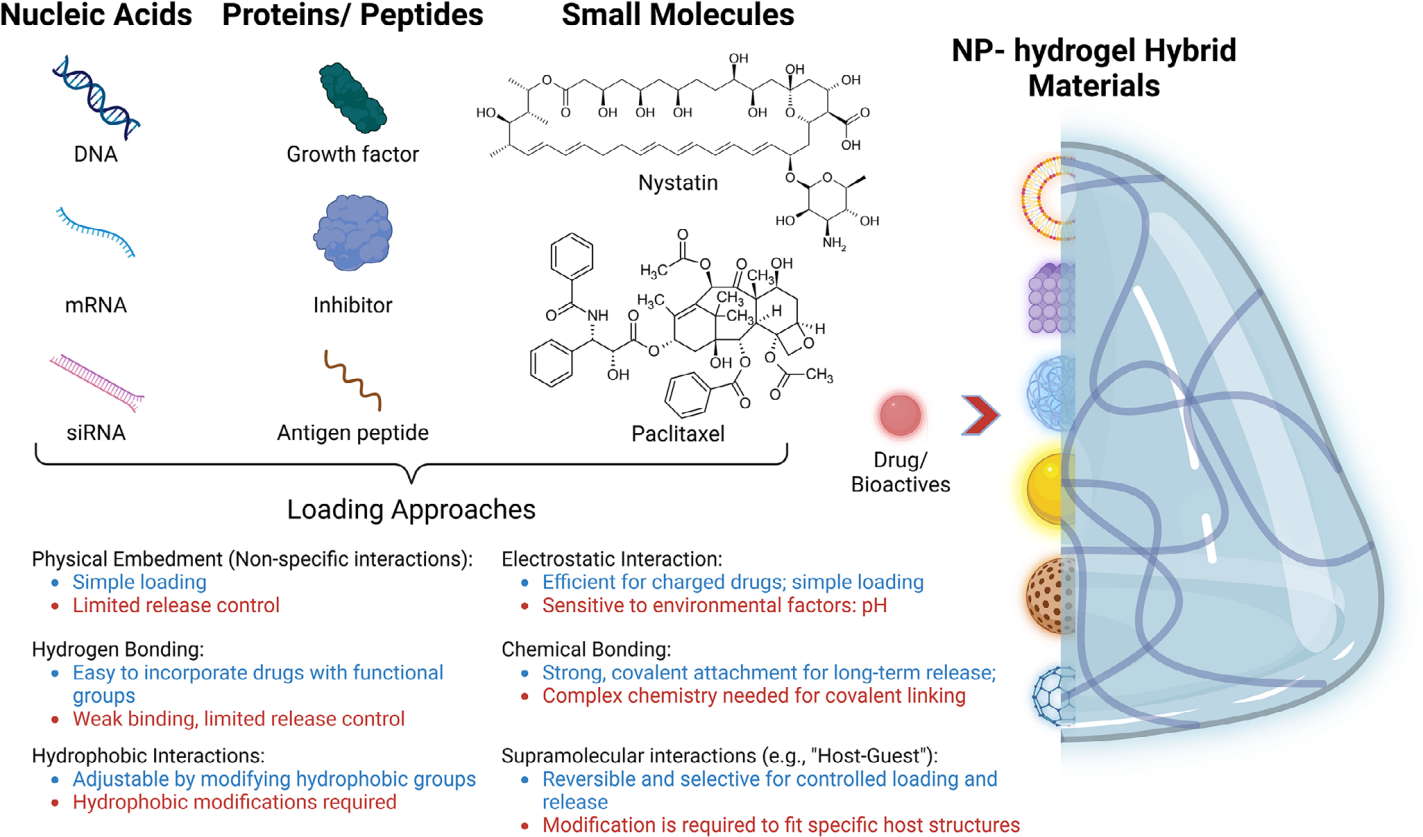
Different classes of drugs and drug loading mechanisms in NP‐hydrogel hybrid materials. The advantages of loading mechanism with different interactions between drug and NPs, or between drug and hydrogels are shown in blue, while the drawbacks are shown in red.

In most NP‐hydrogel hybrid materials used for drug delivery, the therapeutic effect is exerted by the loaded drugs and/or NPs rather than the hydrogel polymers, therefore, the loading mechanism of the drug and NPs into the composite network is a critical aspect defining release behavior.^[^ ^67^, ^139^, ^140^ ^]^ Herein, the loading of drugs into NP nanocarriers or directly into hydrogel networks is discussed first. A range of bioactive or therapeutic agents, such as small molecule drugs (chemotherapeutics, antimicrobials), and biomolecules (proteins, peptides, nucleic acids) have been utilized in the developed hybrid systems (**Figure** **3**), and they are either encapsulated within NPs then introduced into the hydrogel network^[^ ^141^ ^]^ or directly added to the hydrogel network along with NPs.^[^ ^142^ ^]^

Different mechanisms of drug loading have been explored, including physical embedment through nonspecific interaction, hydrogen bonding, hydrophobic interactions, electrostatic interactions, chemical bonding, and supramolecular interactions (Figure 3). The selection of mechanisms for drug loading is constrained to various factors, such as the inherent property of the drug, the NPs, the intended release mechanism and the application.^[^ ^142^, ^143^, ^144^ ^]^ For example, large biomolecule drugs are fragile so that harsh chemical binding and reaction conditions should be avoided to maintain their biofunctionality. The drugs can be loaded via passive absorption,^[^ ^141^ ^]^ hydrophobic interaction,^[^ ^58^, ^98^, ^102^, ^119^, ^121^, ^136^ ^]^ or electrostatic interaction for charged drugs, such as siRNA^[^ ^108^ ^]^ and proteins.^[^ ^145^ ^]^ On the other hand, small molecule drugs for cancer therapy are mostly hydrophobic, which means that hydrophobic loading into NP's hydrophobic cores or membranes of LNPs would be the preferred loading mechanism.^[^ ^146^, ^147^ ^]^

#### 2.2.1 Physical Embedment

Physical embedment via nonspecific interaction involves the passive incorporation of the drug into the interior of NPs or hydrogel polymer network, The drugs are mixed with hydrogel polymer solution before gelation,^[^ ^58^ ^]^ or absorbed into NPs^[^ ^108^, ^120^ ^]^ and hydrogel.^[^ ^116^, ^135^ ^]^ This method is attractive as it is simple and convenient to operate but has several limitations. For loading drugs by mixing drugs with hydrogel polymer solutions, the loading capacity is limited by the water solubility of the drug, which make it only suitable to load hydrophilic drugs into the hybrid system, and the precipitation of hydrophobic drugs may significantly compromise the therapeutic effects.^[^ ^148^ ^]^ For loading drugs to NPs and to pre‐made hydrogels with passive adsorption, it is often limited by the diffusion of the drug and the physical structure of the NPs and hydrogel so in order to increase the amount of loaded drugs, loading time is extended and becomes time consuming. Another approach to improve passive adsorption is to increase the pore size of NPs and pre‐made hydrogels. Larger pores, like those in mesoporous silica NPs^[^ ^120^, ^149^ ^]^ and hydrogel beads with silica‐etching,^[^ ^135^ ^]^ can improve loading efficiency by creating more space for drug diffusion and accommodating more drug.

#### 2.2.2 Hydrophobic Interaction

Hydrophobic interaction is a crucial mechanism for encapsulating hydrophobic drugs into NPs, where the hydrophobic domain of drugs and NPs spontaneously cluster and decreases the overall free energy in the system.^[^ ^150^, ^151^, ^152^ ^]^ Amphiphilic molecules such as lipids and block copolymers are commonly used for loading hydrophobic drugs. The hydrophobic membrane or the core of the NPs can load a relatively large amount of hydrophobic drug and improve solubility, stability, and bioavailability, which is not achievable in hydrogel delivery system alone due to the high water content and the hydrophilic nature of the polymer network.^[^ ^150^, ^151^, ^152^ ^]^

More interestingly, the hydrophobicity can be altered by pH change, such as protonation and deprotonation of amino groups in self‐assembled peptide‐based hydrogel^[^ ^153^, ^154^ ^]^ and ionic shielding effects of ionized polymers.^[^ ^96^ ^]^ By leveraging the pH‐triggered changes in material hydrophobicity, the optimal pH conditions for maximizing drug loading and release can be determined, and pH‐triggered controlled release can be achieved.

#### 2.2.3 Supramolecular Interaction

Supramolecular interactions or “host–guest” interactions involve noncovalent binding between a host molecule, which provides a cavity, and a guest drug molecule that fits within it. Cyclodextrin (CD), a cyclic oligosaccharide, is commonly used for this purpose, with different types (α‐, β‐, γ‐CD) offering varying cavity sizes to accommodate hydrophobic drugs.^[^ ^155^ ^]^ The hydrophobic interior of CDs and hydroxyl groups at the hydrophilic exterior allow for diverse interactions, including hydrophobic interaction, hydrogen bonding, π–π stacking interactions, van der Waals and other weak forces.^[^ ^156^ ^]^ Host–guest interactions are versatile and strong but require tailored modifications for the drug to fit securely within the host unit. For example, Xu et al., enhanced antigen peptide stability and immune cell stimulation by integrating PEG‐modified antigen peptide to Au NPs via thiol affinity binding and forming a supramolecular hydrogel via host–guest interactions between the PEG group and α‐CD.^[^ ^110^ ^]^ Moreover, the guest and/or host unit can be modified with stimuli‐responsive chemical groups to obtain a specific responsiveness, Yuan et al., encapsulated siRNA and DOX‐loaded mesoporous silica NPs (MSNs) in a pH and redox dual‐responsive CD hydrogel via “host–guest” interactions between β‐CD and dithiopyridine unit on MSN. The interaction can be weakened at acidic pH where the nitrogen in the dithiol‐pyridine ring is protonated, resulting in the formation of a pyridinium and reducing the hydrophobicity of the pyridine. On the other hand, the dithiol linkage can be cleaved with the presence of Glutathione (GSH) and release the encapsulated drug.^[^ ^108^ ^]^

#### 2.2.4 Electrostatic Interaction

Electrostatic interactions provide a straightforward and effective method for loading charged drugs by tuning the surface charge of NPs to attract oppositely charged drugs. For instance, in the nanodelivery system developed by Yuan et al., negatively charged siRNA was coloaded with positively charged units on MSNs through electrostatic forces, reducing drug leakage.^[^ ^108^ ^]^ NP surface charge can also be modified by adjusting pH, which influences the strength of electrostatic interactions and provides pH‐responsive controlled release in certain environments. For example, Yang et al., loaded negatively charged siRNA to positively charged Polyethylenimine (PEI) NPs via electrostatic interaction and synthesized a pH‐sensitive NP‐hydrogel hybrid material that prolonged siRNA release in acidic release solution.^[^ ^157^ ^]^

#### 2.2.5 Chemical Bonding

Chemical bonding involves the formation of strong chemical bonds which can covalently load drugs to nanocarriers, providing strong and stable attachment and allowing for controlled, prolonged release independent of carrier degradation. Drugs can also be loaded via cleavable chemical linkers, such as ester, acetal, disulfide, thioether, nitrobenzyl, and cyanine, which are designed to break at certain physicochemical conditions, including pH, light, temperature, reactive oxygen species (ROS), and specific enzymes.^[^ ^46^, ^158^, ^159^ ^]^ For example, Liu et al., precisely immobilized one bismuth selenide nanosheet on each dextran NP by forming the Schiff base bond via crosslinking with glutaraldehyde to integrate a photothermal property to a NP‐hydrogel hybrid material.^[^ ^124^ ^]^ Another example is that brain‐derived neurotrophic factor was loaded into amine‐functionalized titanium oxide NPs via covalent amide and thioether bonds. The chemical linkage prevents the potential leakage of the growth factor drug from NPs and the growth factor is release with bound NPs without UV exposure. Due to the UV‐activation property of titanium oxide NPs, the growth factor can be released from NPs with UV activation, and attached to hydrogel fiber with strong affinity, which reduces the overall release of growth factor and controls the release profile with UV exposure.^[^ ^134^ ^]^ While chemical bonding enables precise control over drug loading quantity and release, it can be costly and time consuming for modifications, with potential drug deactivation and low yield during synthesis.^[^ ^108^, ^110^, ^155^, ^156^ ^]^

### 2.3 NP Loading in Hydrogels

**Figure 4:**
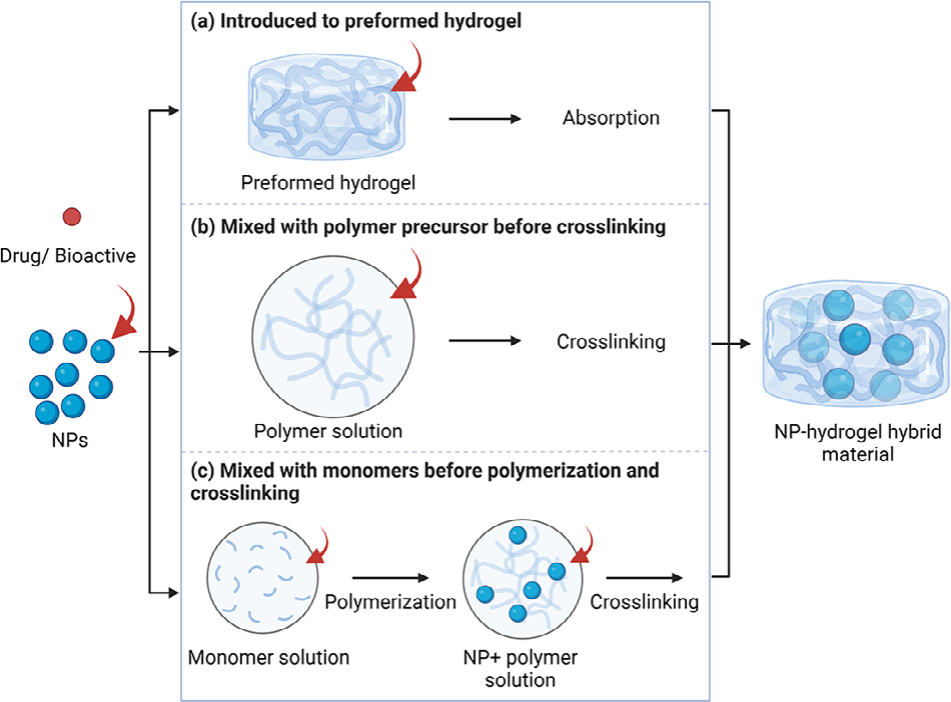
Three routes for incorporating NPs in hydrogel networks. a) NPs are introduced to preformed hydrogel. b) NPs are mixed with polymer solution before crosslinking into hydrogel. c) NPs are mixed with monomers before polymerization and crosslinking to hydrogels.

Incorporation of NPs into hydrogel networks can be achieved through different routes,^[^ ^160^ ^]^ including loading to the preformed hydrogel (**Figure** **4**a), mixing with polymer solution before gelation into the hydrogel (Figure 4b) and mixing with monomer solution followed with polymerization and crosslinking to form the hydrogel (Figure 4c). There are several previously published reviews already summarizing different approaches to synthesize NP‐hydrogel hybrid materials.^[^ ^67^, ^144^ ^]^ The interaction between NPs and the hydrogel network is highly governed by physical forces, such as van der Waals interactions, electrostatic forces, and/or hydrogen bonding.^[^ ^161^ ^]^ Furthermore, the surface chemistry of the NPs can be modified to have affinity toward specific chemical groups of the polymer chains in the hydrogel, leading to stronger interaction, enhanced stability, and tunable release kinetics.^[^ ^161^, ^162^ ^]^

The loading of NPs into preformed hydrogels (Figure 4a) primarily relies on the diffusion of NPs into the porous network of the hydrogel matrix upon incubation, This process is influenced by the hydrogel pore size, NP particle size and surface characteristics of NPs.^[^ ^160^ ^]^ However, it may suffer from poor NP distribution due to weak interactions and low penetration of NPs through the entire hydrogel structure.^[^ ^117^ ^]^ By mixing the NPs with the polymer solution before cross‐linking to the final hydrogel (Figure 4b), a higher NP loading and a more uniform distribution of NPs within the hydrogel can be achieved. This method is advantageous as it allows the NPs to be incorporated homogeneously throughout the hydrogel network, improving the mechanical strength and functional properties of the hybrid material.^[^ ^97^ ^]^ For example, Liu et al., designed a NP‐hydrogel hybrid material composed of carrageenan hydrogel and Ag NPs coated with gallic acid, which combined the photothermal effect of gallic acid and the antibacterial property of Ag NP for improved antimicrobial effects.^[^ ^163^ ^]^ NPs that have surface functional groups compatible with the polymers can form secondary interactions, such as hydrogen bonding or ionic interactions, which enhance their integration and stability within the hydrogel.^[^ ^93^, ^162^ ^]^ For example, Tao et al., incorporated copper NPs in a hydrogel of gelatin methacrylate (GelMA) and *N*,*N*‐bis(acryloyl) cystamine (BACA), where the disulfide bonds on BACA stabilized copper NPs from oxidation and improved their stability and sustained release.^[^ ^164^ ^]^

In a third method, NPs are dispersed with monomer solution and therefore present during the polymerization procedure (Figure 4c).^[^ ^165^ ^]^ This mechanism ensures a high degree of embedment for the NPs within the hydrogel and prevents leaching during application. During the process, NPs can be chemically trapped within the growing polymer network, further anchoring them to the hydrogel matrix which also reinforces the mechanical strength of the hydrogel.^[^ ^166^ ^]^ For example, NP‐hydrogel hybrid material was synthesized by Li et al., via in situ copolymerization of poly(2‐acrylamido‐2‐methylpropane sulfonic acid‐*co*‐*N*, *N*‐dimethylacrylamide), while embedded NPs (graphene oxide and Laponite) served as additional crosslinking agents and enhanced the ultrastretchability of the composite material.^[^ ^167^ ^]^ Similarly, hybrid material was synthesized by Manjusha et al., by crosslinking chitosan coated iron oxide NPs with [2‐(methacryloyloxy) ethyl] trimethyl ammonium chloride (METAC) via radical polymerization. The combination of NPs and METAC hydrogel enabled the specific targeting of human breast cancer cells and improved the release of PTX by over 90% at pH 5.5 over 24 h.^[^ ^96^ ^]^ Additionally, the NP precursors are added to the monomer/polymer solutions and the NPs are synthesized during the polymerization and gelation process. For example, Nasef et al., incorporated Ag and Au NPs by mixing the silver nitrate and gold (III) chloride with acrylamide and reducing Ag and Au ions to zero valence Ag and Au NPs during the gamma irradiation polymerization of polyacrylamide. The hybrid material of Ag/Au NPs with polyacrylamide was reported to reduce the cytotoxicity of Ag NPs while enhancing their effectiveness in suppressing liver cancer cell growth.^[^ ^117^ ^]^ Similarly, Khalil et al., incorporated Ag NPs to chitosan/polyacrylamide hydrogel by converting Ag^+^ to the Ag NPs during the gamma irradiation polymerization of chitosan and acrylamide. The hybrid material showed advantages in antifungal and anticancer applications.^[^ ^165^ ^]^

## 3 Key Properties of the Hybrid Materials Affecting Drug Release and Delivery

Smart design of NP‐hydrogel composite materials as drug delivery systems requires an understanding and characterization of the key properties of the final composites, including the porous structure and pore size, the mechanical strength, and stability, These properties can be greatly impacted by incorporating NPs into a hydrogel depending on the type of interactions between the NPs and the hydrogel matrix, which subsequently influence drug release.^[^ ^168^ ^]^ These properties of NP‐hydrogel hybrid materials are inter‐related in that drug diffusion rate is primarily affected by the porous structure and pore size of hydrogel, while pore size and porosity have also significant impacts on material mechanical strength, The mutual connection between these properties is relatively under explored.

Drug release by diffusion is usually modeled with Fickian Diffusion

(1) $$\frac{\partial C}{\partial t}=D_{\mathrm{eff}}\;\frac{\partial^{2}C}{\partial z^{2}}$$

where the *C* is the drug concentration and *D*_eff_ is the effective diffusion coefficient, which is dependent on the diffusion medium. For drug diffusion in solution, such as phosphate‐buffered saline (PBS) and cell culture medium, the diffusion coefficient is given by the Stokes−Einstein equation

(2) $$D_{0}=\frac{k_{b}T}{6\pi \eta r}\;$$

where *k*_b_ is the Boltzmann constant, T the temperature, *η* is the medium viscosity, and *r* is the hydrodynamic radius of the diffusing drug.^[^ ^169^ ^]^ The overall drug release rate is limited by the drug diffusion rate from hydrogel for drug delivery with hydrogel system due to the porous structure of hydrogel,^[^ ^170^ ^]^ and the effective diffusion coefficient through hydrogel is described as

(3) $$\frac{D_{\mathrm{eff}}}{D_{0}}=\exp\left(-\pi {\left(\frac{r+r_{f}}{\xi +2r_{f}}\right)}^{2}\right)$$

where *r_f_* is the polymer chain radius and *ξ* is the mesh size,^[^ ^171^ ^]^ which highlights the relevance of hydrogel pore size and porosity on controlling drug release. On the other hand, porosity may compromise the overall strength, classically the elastic modulus of porous materials can be described with the Gibson and Ashby model

(4) $$\frac{E}{E_{s}}=\;C{\left(1-\varphi_{p}\right)}^{n}$$

where 

(5) $$\varphi_{p}=1-\frac{\rho }{\rho_{s}}$$

where *E* and *E*_s_ are the elastic modulus of the porous material and the solid nonporous material, respectively, and *ρ* and *ρ*_s_ are the density of porous and nonporous materials, respectively, with *φ*_p_ is the porosity of the porous material.^[^ ^172^ ^]^ The mechanics of hydrogels can be further contextualized as swollen polymer networks considering rubber elasticity, swelling, and mesh size, e.g.,^[^ ^173^, ^174^ ^]^ and the Young's modulus (*E*) can be described by the strand elasticity (*k*) and strand density (*v_x,_*_0_ *λ*_s_^−3^) as *E = kv_x,_*_0_ *λ*_s_^−3^.^[^ ^68^ ^]^

Incorporation of NPs in hydrogel network introduces extra molecular interactions between NPs and polymers in hydrogel, which alters the spatial organization of NPs and polymer chains, enhances mechanical strength, affects porous structure, and eventually influences drug release, Ghandforoushan et al., incorporated TGF‐β1‐loaded PLGA‐PEG‐PLGA nanoparticles in PLGA‐collagen hydrogel for cartilage tissue engineering.^[^ ^175^ ^]^ The average pore size and porosity decreased from 133 µm and 61.3% to 100 µm and 42.6% after adding PLGA‐PEG‐PLGA nanoparticles in PLGA‐collagen hydrogel, respectively, while the material density and Young's modulus increased from 0.135 g cm^−3^ and 0.58 MPa to 0.184 g cm^−3^ and 0.81 MPa, respectively, resulting in a more condensed polymer mesh with reduced swelling and slower degradation. Moreover, other than the initial burst release of TGF‐β1 from PLGA‐collagen hydrogel, the incorporation of NPs controlled the TGF‐β1release in a sustained and prolonged manner over 21 days with a final cumulative release of 78%.^[^ ^175^ ^]^

### 3.1 Porous Structure

The structural properties of the hydrogel depicted by the polymer crosslinking density and pore size or mesh size of the 3D network, are critical for controlling water absorption, swelling, stability, drug‐, or NP‐loading efficiency.^[^ ^176^, ^177^, ^178^ ^]^ More importantly, the porous structure of the hydrogel directly influences the release kinetics of physically embedded drugs or NPs.^[^ ^143^ ^]^ Lower polymer crosslinking density and larger pore size can lead to faster diffusion of NPs or drugs out of the system while higher density and more viscous hydrogel with small mesh size slows down the diffusion.^[^ ^174^, ^179^ ^]^

**Figure 5:**
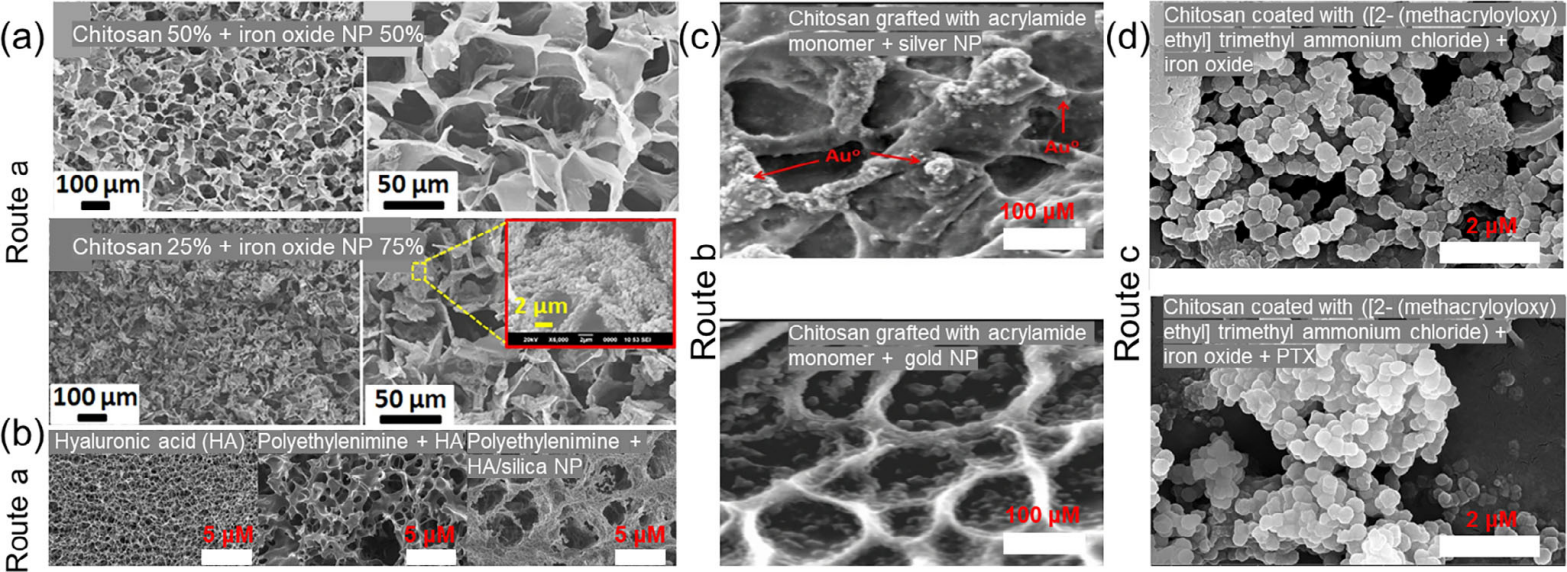
Porous structures of NP‐hydrogel hybrid materials prepared by the three routes. a) Iron oxide NPs in chitosan hydrogels showing a densified structure with increased NP content. Adapted with permission.^[^ ^60^ ^]^ Copyright 2022, Elsevier. b) Silica NPs in HA/PEI hydrogel where a dense SiNP network with micrometer‐ and nanoscale pores was observed. Adapted with permission.^[^ ^59^ ^]^ Copyright 2022 under CC BY 4.0 license, Elsevier. c) Silver and Au NPs in chitosan hydrogel via polymer gelation with spherical NPs enhancing antibacterial and pH‐responsive drug release. Adapted with permission.^[^ ^117^ ^]^ Copyright 2023 under CC BY 4.0 license, Springer Nature. d) Iron oxide NPs in chitosan hydrogel via monomer polymerization. Adapted with permission.^[^ ^96^ ^]^ Copyright 2023, Elsevier.

In some cases, the structure of the NP‐hydrogel composites can be significantly impacted by the stronger interactions between the embedded NPs and the polymers of the hydrogel, especially those prepared via covalent chemical bonds, hydrogen bonds and electrostatic interactions,^[^ ^180^ ^]^ thus enhancing drug loading, NP embedment and controlled release. For example, the charge interactions between Ag‐doped MSN embedded in the network of a PEG diacrylate/HA hydrogel decreased the overall pore size (from average pore size of 238.1–110.7 µm) and hydrogel swelling, while an increase in mechanical strength and adhesion strength and a more stable and controlled release of Ag over 3 weeks were observed.^[^ ^127^ ^]^ The MSN‐PEG hydrogel hybrid material was used as a wound dressing, responded to the acidic environment of infected wounds, enabled controlled Ag NPs release for sustained antibacterial activity while promoting cytocompatibility and angiogenesis. A similar effect was also observed by Sumitha et al., when introducing magnetic iron oxide NPs to a chitosan based hydrogel.^[^ ^60^ ^]^ **Figure** **5**a shows the inter‐related pore structure of the iron oxide NP‐chitosan hydrogel composite.^[^ ^60^ ^]^ A densified surface texture was noted with the addition of more iron oxide NPs when the NP content increased from 50% to 75% (Figure 5a), which consequently contributes to the increased compressive strength and hence allows the system to endure internal mechanical loads for targeted cancer therapy by on‐demand magnetically triggered drug delivery and hyperthermia. The porous structure was affected by the different orientation of NPs in the hydrogel network under a magnetic field, and the expansion and contraction of the porous network promotes drug release due to fast diffusion. Newham et al., prepared a HA hydrogel with PEI functionalized silica NPs (SiNP), where the electrostatic interactions between positively charged PEI and negatively charged HA led to a densely packed SiNP network in the hydrogel featuring micrometer‐scale and nanoscale pores (Figure 5b). Increasing the SiNPs to HA polymer ratio resulted in a decreased overall pore size and a decreased and more sustained Methotrexate (MTX) drug release rate.^[^ ^59^ ^]^ The HA hydrogel alone showed a burst release where more than 80% of MTX was released within 8 h, and nearly 100% was released over 24 h, while the hybrid materials with a 3.6/4.8 PEI‐SiNP/HA ratio provided 47% MTX release in 8 h, 68% MTX release in 24 h, and sustained increase over 72 h.^[^ ^59^ ^]^ Nasf et al., observed the scanning electron microscope image of a pH‐responsive nanocomposite hydrogel of chitosan grafted with acrylamide monomer and Ag and/or Au NPs, and found the spherical NPs were in the pores and on the surface (Figure 5c).^[^ ^117^ ^]^ This hybrid material combined the antibacterial effect of Ag NPs, biocompatibility of Au NPs with the pH‐responsive chitosan/polyacrylamide (Cs‐g‐PAAm) hydrogel for oral administration of an anticancer drug Fluorouracil. Decreased hybrid material swelling and a decreased drug release rate were observed when incorporating Ag NPs into the hydrogel, while the contrary effects were observed when incorporating Au NPs into the hydrogel where the optimal drug release of 33%, 87%, 22%, and 97% was achieved over 315, 450, 375, and 300 min for Cs‐g‐PAAm hydrogel, Cs‐g‐PAAm/AuNPs, Cs‐g‐PAAm/AgNPs, and Cs‐g‐PAAm/Au–Ag‐NPs hybrid materials, respectively.^[^ ^117^ ^]^ Manjusha and coworkers loaded iron oxide NPs into a chitosan hydrogel.^[^ ^96^ ^]^ They found that the NP‐chitosan composite hydrogel has a smooth surface with cavities facilitating drug diffusion into the hydrogel network, while the well‐packed, smooth surface of the drug loaded hydrogel confirms successful PTX loading (97.1% drug loading efficiency) (Figure 5d), thus achieving efficient hydrophobic drug delivery and enabling a pH triggered PTX drug release.

### 3.2 Stability and Mechanical Strength

In general, as described before, mechanical properties of a hydrogel can affect the porous structure and thus affect drug release in which a strong and viscous hydrogel slows passive diffusion of the drug or NPs. On the contrary, a weak hydrogel may be prone to degradation and premature loss of the crosslinked structure inevitably leads to release of the embedded drug or NPs. The overall mechanical strength, stability, and degradation properties of NP‐hydrogel hybrid materials depend on both the polymer matrix and the interaction between the matrix and the embedded NPs. Photocrosslinked polymers, such as GelMA, HA‐*o*‐nitrobenzene, and HA‐carbohydrazide, have been used to incorporate NPs in hydrogel system with strong molecular interactions, form a strong hydrogel with stable crosslinkers and enhanced mechanical strength.^[^ ^181^ ^]^ For example, Zhang et al., discovered hydrogels of HA‐*o*‐nitrobenzene and HA‐carbohydrazide effectively retain and protect PLGA NPs loaded with therapeutic agents, facilitating wound healing while being biodegradable and biocompatible.^[^ ^98^ ^]^ The PLGA NPs were grafted with *o*‐nitrobenzene, photoinitiated crosslinked with hydrogel network, and the hybrid material storage modulus and compression modulus increased from 270 Pa and 14.6 kPa to 350 Pa and 19.1 kPa for hydrogel alone and hydrogel with 6% weight of PLGA NPs, respectively, The incorporation of PLGA NPs enhanced the mechanical strength of hybrid materials, prolonged the release of TGFβ inhibitor from 1 day to 8 days, provided a sustained release of bioactive and improved wound healing and scar suppression.^[^ ^98^ ^]^ On the other hand, embedded NPs themselves often can enhance the mechanical properties of hydrogel composites^[^ ^59^, ^182^ ^]^ and widen the biomedical applications which require a more stiff or a more durable scaffold.^[^ ^183^, ^184^ ^]^ For instance, matrix stiffness is essential in bone tissue generation^[^ ^66^, ^185^ ^]^ and carbon NPs were embedded in alginate hydrogel to improve bone cell growth and bone reconstruction.^[^ ^186^ ^]^ Moreover, a stronger interaction between NPs and hydrogel matrix can be created via the hydrogen bonding. For example, the hydrogen bonds between MXene and agarose hydrogel significantly enhanced the mechanical strength and durability of the hybrid material, enabled a NIR‐triggered drug release and prevented potential drug and NP leakage from the hydrogel without NIR exposure.^[^ ^112^ ^]^There was no obvious DOX release from the hybrid material with absence of NIR, but the drug release was significantly promoted and reached a maximum release of 80% in 24 h with NIR illumination.^[^ ^112^ ^]^

## 4 Biocompatibility, Bio‐adhesion and Self‐Healing Properties

Biocompatibility is a key factor in drug delivery systems, directly impacting therapeutic efficacy and patient safety. Biocompatibility is typically quantified by in vitro cell‐viability assays, with acceptable systems showing ≥ 70%–80% viability at relevant NP concentrations, and best‐in‐class materials maintaining > 90% viability even at > 100 µg mL^−1^ according to the ISO 10993‐5 standard.^[^ ^187^ ^]^ The cytotoxicity of NPs is relevant to their clinical applications. The toxicity of NPs is dependent on various parameters including size, shape, surface charge, chemistry, and surface modification.^[^ ^188^ ^]^ NPs, especially metal‐based NPs, have high surface area to volume ratio, which leads to high surface chemical reactivity and ROS generation and the high ROS stress can result in cytotoxicity and consequent damage to healthy cells and tissue.^[^ ^188^, ^189^, ^190^ ^]^ With the hydrogels, the cytotoxicity of NPs and hydrogels must be carefully assessed to ensure that their integration does not elicit adverse biological responses, Composite materials often show improved biocompatibility due to the synergistic effects of their components. It has been shown that the inclusion of biocompatible polymers like PEG and PLGA can mitigate the potential toxicity of metallic NPs,^[^ ^191^, ^192^, ^193^ ^]^ making the composites suitable for biomedical applications, such as cancer treatment and wound healing. For example, Zhang et al., developed GelMA hydrogel embedding Zeolitic imidazolate framework‐8@ceric oxide NPs to deliver DOX for tumor postoperative treatment and wound repair.^[^ ^131^ ^]^ The hybrid material showed improved tumor suppression and wound healing while maintained relatively high cell viability for L929 and MCF‐10A cell lines.^[^ ^131^ ^]^

Moreover, since NP toxicity increases with concentration,^[^ ^194^, ^195^ ^]^ incorporating NPs into hydrogel networks helps modulate their local concentration by controlling NP release, retention, and distribution, thereby maintaining levels within an effective biocompatible and therapeutic range. A recent study developed an injectable, self‐healing hydrogel composed of carboxymethyl chitosan and aldehyde‐functionalized dextran, incorporating in situ synthesized Ag NPs for enhanced wound healing applications.^[^ ^196^ ^]^ The hydrogel demonstrated a controlled release of silver ions, with ≈35% released over 48 h in phosphate‐buffered saline (PBS) at 37 °C. This sustained release profile significantly reduced cytotoxicity compared to free Ag NPs. In vitro cytocompatibility assessments using L929 fibroblast cells showed over 85% cell viability at an equivalent Ag NP concentration of 20 µg mL^−1^ after 24 h, indicating excellent biocompatibility for the hydrogel system.^[^ ^196^ ^]^

Bioadhesion refers to the ability of a material to adhere to biological tissues, which is a crucial property for localized drug delivery. Effective bioadhesion ensures that the drug delivery system remains at the target site, optimizing drug release profiles and minimizing systemic dispersion.^[^ ^197^ ^]^ In the context of NP‐hydrogel composites, bioadhesion is particularly important in applications like wound healing, where adherence to the wound site can enhance the delivery of antimicrobial agents and support tissue regeneration.^[^ ^124^ ^]^ The study by McCrorie et al., exemplifies this, where pectin‐based hydrogels demonstrated excellent adhesion to brain tissue, facilitating sustained drug release and promising localized treatment for brain cancer.^[^ ^198^ ^]^ While effective adhesion to tissue surfaces is quantified by lap‐shear or peel tests, with robust adhesives achieving 5–20 kPa and specialized systems reaching > 100 kPa, a recent review shows that π–π and cation–π interaction hydrogels (incorporating aromatic and cationic monomers) can achieve an exceptional adhesion strength of ≈180 kPa on steel substrates, illustrating the upper range for bio‐inspired adhesives.^[^ ^199^ ^]^ Liang et al., showed a polydopamine nanoparticle‐reinforced double network hydrogel (PAM‐alginate) and achieved a lap‐shear strength of 146.84 ± 7.78 kPa on glass after 14 days in seawater, highlighting the significant enhancement of adhesion through nanoparticle incorporation.^[^ ^200^ ^]^

Self‐healing properties in hydrogel composites allow them to recover their structure and function after physical damage, which is advantageous in dynamic biological environments. This property ensures the longevity and durability of the drug delivery system, especially in scenarios where the hydrogel might be subjected to mechanical stress. For instance, injectable Schiff base hydrogels containing amine‐modified silica NPs not only exhibit rapid self‐healing but also show tailored degradation responsive to environmental pH changes, thereby offering controlled drug delivery.^[^ ^201^ ^]^ Some works have quantified the self‐healing efficiency by the recovered fraction of original mechanical strength after damage, and hydrogels commonly fall between 70% and 100% within s. For example, carboxymethyl chitosan–oxidized‐cellulose hydrogel with bioactive‐glass NPs displayed ≈82% healing efficiency (fracture stress recovery) over 24 h, owing to dynamic Schiff‐base bonds.^[^ ^202^ ^]^ Liu et al., developed a gold nanoparticle–selenium nanocomposite hydrogel based on Au–Se coordination within a polyacrylamide matrix and demonstrated a rapid self‐healing efficiency of 91% within 2 min under NIR irradiation, along with excellent mechanical properties including a fracture stress of 2.48 MPa and elongation at break of 1970%, highlighting the role of nanoparticle‐induced dynamic interactions in enabling multiresponsive self‐repair.^[^ ^203^ ^]^

## 5 Mechanisms of Drug Release of the Hybrid Materials

**Figure 6:**
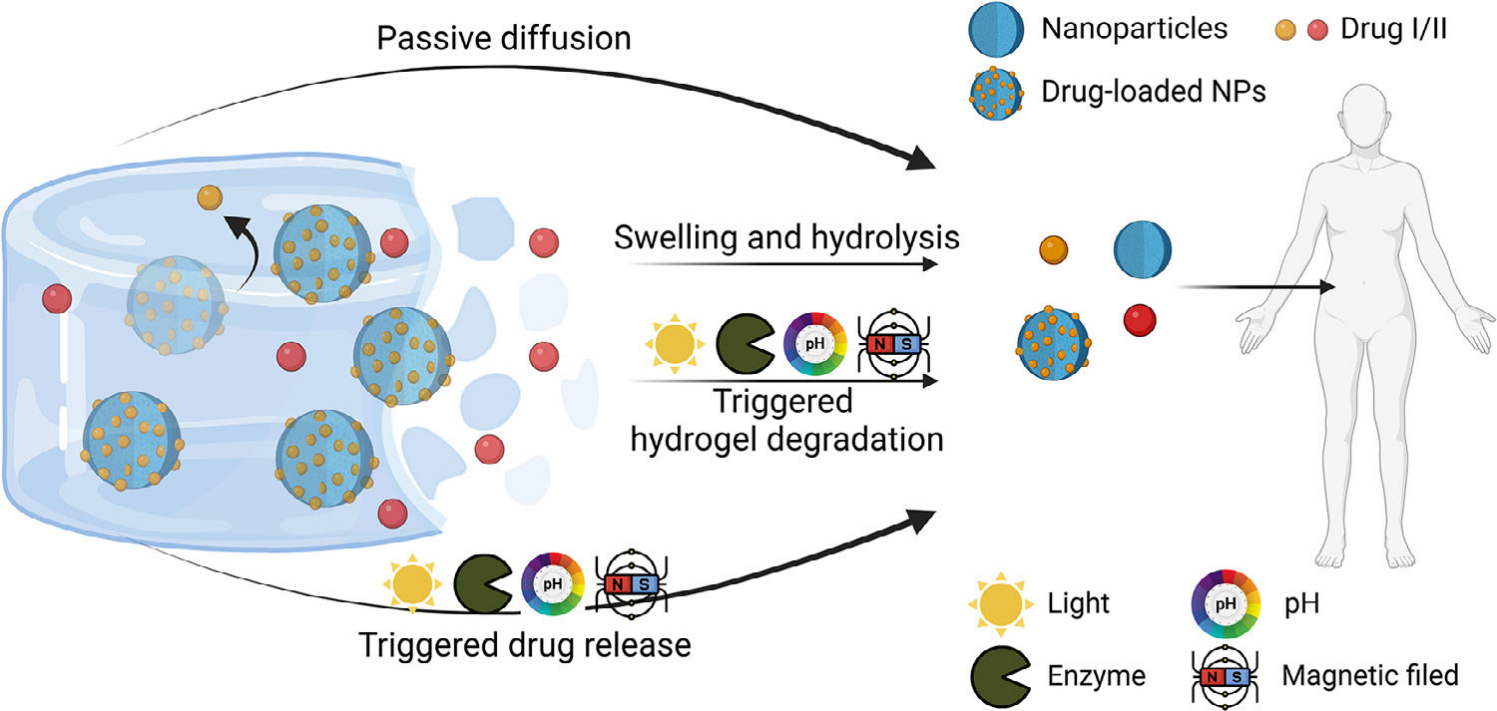
Drug release mechanisms of NP‐hydrogel hybrid materials.

One of the most promising aspects of bioactive‐loaded NP‐hydrogel hybrid materials is its ability to load a higher amount of therapeutic agents and better control release than either NP or hydrogel drug delivery systems alone^[^ ^204^, ^205^, ^206^ ^]^ and to prevent uncontrolled burst release and drug leakage.^[^ ^205^ ^]^ Numerous studies have demonstrated that by combining the unique advantages of the NP and hydrogel systems, high local retention of administered drugs, multifunctioning and specific stimuli‐controlled drug release can be achieved.^[^ ^142^, ^207^, ^208^ ^]^ **Figure** **6** illustrates various mechanisms of drug release which can occur in a NP‐hydrogel hybrid system, and due to the complexity of the materials, simple passive diffusion, triggered‐release from NPs responsive to stimuli, hydrolysis‐induced hydrogel breakdown, as well as triggered‐release from the degradation of hydrogel polymer network responsive to stimuli, are all possible release routes which can be exploited by researchers with end applications in mind. These strategies are achieved by tailoring the NPs’ and hydrogels’ chemical, structural, and physical properties, such as adjusting its drug‐affinity, degradation rate and mechanical features, as well as controlling the interaction between drugs, the NPs, and the hydrogel network. Additionally, external stimuli, such as temperature, light irradiation, pH, enzymatic reactions, and magnetic fields can trigger responsive NP or hydrogel polymer behavior, ensuring on‐demand release at cancerous tumors, infected sites, or damaged tissues.^[^ ^209^, ^210^, ^211^, ^212^ ^]^ This section presents the drug release behavior of the composite material from the perspective of both the embedded NPs and the macroscopic hydrogel polymer network.

In many cases, hydrogel polymer networks are nonresponsive, and drugs can be released out of the hydrogel and NPs through passive diffusion or stimuli‐induced mechanism. On the other hand, cross‐linked polymer networks can undergo hydrolysis or respond to stimuli, leading to hydrogel degradation accelerating the release of the embedded drugs and NPs.

### 5.1 Passive Diffusion

Passive diffusion in drug delivery is the most common mechanism of drug release in which drug molecules simply diffuse out of the carriers due to concentration gradient without any external stimuli and passes through biological media to exert a therapeutic effect. The diffusion rate is limited by a number of factors including the chemical nature and size of the drug molecule, the physicochemical property and the structure of the drug carrier systems, and the rheology of the biological media. In a NP‐hydrogel composite drug delivery system, the diffusion process of the encapsulated drug is further determined by the location and embedding mechanism of the drug within the system.^[^ ^213^, ^214^ ^]^ As discussed above, hydrophilic drugs can be directly embedded within the crosslinked hydrogel network and in this scenario, diffusion is influenced by the pore size, shape, tortuosity, and swelling behavior of the hydrogel. Raza et al., loaded hydrophilic Nystatin into a chitosan/HA hydrogel and reported that varying the ratio of chitosan/HA can influence the swelling of the polymer network and the drug release. Specifically, the optimal formulation (1% chitosan and 0.02% HA) had 87.5% swelling and 90% of Nystatin release in 12 h, while another formulation (0.5% chitosan and 0.4% HA) had 37.5% swelling and 71% release.^[^ ^111^ ^]^

Encapsulating drugs in NPs which are then embedded into the hydrogel can significantly slow down the diffusion as they need to be released from the NPs and then cross the hydrogel network, or the NPs, often much larger in size, need to diffuse out of the hydrogel network first before releasing the encapsulated drugs. For instance, proteins directly encapsulated in hydrogels usually experience a burst release.^[^ ^215^, ^216^ ^]^ To address this issue, Zhang et al., designed a PLGA NP‐HA hydrogel hybrid system and found that encapsulation of a protein drug, i.e., the growth factor‐β inhibitor within the composite system led to a delayed release of the protein than directly loading them into the hydrogel, and this controlled release can be further prolonged with the increase of PLGA polymer molecular weight.^[^ ^98^ ^]^ Incorporation of NPs can also control the hydrogel swelling rate and drug diffusion. For example, demonstrated by Nasef et al., incorporating Ag NPs decreased the swelling ratio of a chitosan and acrylamide‐based hydrogel by half at pH 7.4, while incorporating Au NPs doubled the swelling, and incorporating both Ag and Au NPs led to a moderate increase of swelling.^[^ ^117^ ^]^ Thus, the swelling behavior of the hybrid material became tunable, which greatly enhanced the loading of a model drug, fluorouracil, by twofold, and improved the drug release from 22% to 97% over 300 min.

### 5.2 Stimuli Triggered Release from Intact and Nonresponsive Hydrogels

Stimuli‐triggered release is designed to release therapeutic agents in response to specific environmental triggers, including light, pH, redox conditions, chemicals, enzymes, and electrical and magnetic fields.^[^ ^217^, ^218^ ^]^ In the composite system, the building block of a hydrogel, such as alginate, HA, and PEG is often nonresponsive to these stimuli so that while the structural integrity of the hydrogel and the hybrid material is maintained, the endogenous or exerted stimuli act on the interaction between loaded drugs and NPs.^[^ ^219^, ^220^ ^]^ Reviewing stimuli‐responsive NP drug delivery systems is out of the scope of this review. However, herein, examples of drug release from NP‐hydrogel hybrid materials triggered by common stimuli acting upon NPs are provided.

#### 5.2.1 Light

Light‐triggered drug release provides significant advantages, including convenience, noninvasiveness, user‐defined remote controllability, temporal, and spatial selectivity as well as precise “on/off” drug release control.^[^ ^221^ ^]^ For example, *o*‐nitrobenzyl is a light‐sensitive functional group which has been used to link a drug to polymer, silica, or metal oxide NPs, and used to modify the NP materials to make the NPs photosensitive.^[^ ^222^ ^]^ Upon shining UV light, the *o‐*nitrobenzyl chemical bond is broken, producing an aldehyde/ketone moiety allowing detachment and subsequent release of the encapsulated drugs.^[^ ^223^, ^224^ ^]^ For example, a DNA inhibitor drug, camptothecin (CPT), was chemically attached to silica NPs via a chemical adaptor (4‐hydroxymandelic acid) with photocleavable *o*‐nitrobenzyl group and then embedded in a PEG‐based hydrogel network.^[^ ^224^ ^]^ The cumulative release of CPT increased from less than 10% (no UV) to about 100% release under 10 min UV exposure over 24 h, indicating a complete breakdown of the linkage and UV triggered CPT release.

Drug release can also be regulated by the degradation of photosensitive NPs^[^ ^223^, ^225^ ^]^ or the detachment from photocatalytic NPs, such as titanium oxide.^[^ ^134^ ^]^ NPs made of lipid and polymer molecules can be modified with photocleavable *o*‐nitrobenzyl groups,^[^ ^223^ ^]^ and the *o*‐nitrobenzyl groups on NPs serve as a hydrophobic segment to increase the hydrophobicity. After UV exposure, the *o*‐nitrobenzyl modified amphiphilic molecules break down to smaller molecules, shift the hydrophobic‐hydrophilic balance and release the encapsulated drug.^[^ ^225^ ^]^ Bruggeman et al., attached a growth factor drug to photoactive titanium oxide NPs, which readily diffused out from the hydrogel with the bonded growth factor without UV irradiation. Once the growth factor was detached from the NPs by UV exposure, it showed higher affinity toward the hydrogel nanofibers and was retained within the hydrogel, reducing its overall release rate, UV exposure thus facilitates a transfer of the growth factor from the NPs to the hydrogel, altering the diffusional release profile, which establishes a more stable release with UV irradiation.^[^ ^134^ ^]^

Photothermal NPs, including metal‐based and carbon‐based NPs, are attractive in cancer therapy due to their capability to effectively convert light irradiation into heat, and kill cancer cells with high temperature.^[^ ^226^, ^227^ ^]^ The heat generated by photothermal effects also impact the hydrogel network, and the mechanism of controlling drug release in composite materials with photothermal NPs will be discussed in Section 4.3.

#### 5.2.2 pH

Deviation from normal tissue pH (homeostatic pH of 7.3–7.4 for most tissue) is often associated with various pathological conditions, such as cancer, infections, tissue damage, and diseases,^[^ ^228^ ^]^ whereby drug release can be achieved upon endogenous pH variation from pH sensitive carriers, pH‐triggered release has been exploited in various kinds of NPs made of pH‐sensitive polymers (PLGA,^[^ ^229^ ^]^ PDMAEMA,^[^ ^230^ ^]^ PEI,^[^ ^231^ ^]^ etc.). These changes disrupt chemical bonds, such as ester, acetal, and Schiff base, accelerate NPs degradation and release the encapsulated drugs.^[^ ^220^, ^232^, ^233^ ^]^ Huang et al., developed a Ag‐doped MSN loaded in a crosslinked HA‐based hydrogel.^[^ ^127^ ^]^ As the Ag‐doped MSN dissolved at mild acidic solution, the NP‐hydrogel composite exhibited Ag release threefold faster at the bacterial‐infected wound site (pH 6.5) than in a neutral pH 7.4 condition.^[^ ^127^ ^]^

Moreover, surface charge of the NPs and mesh size of the polymer hydrogel can be tuned by changing the pH, affecting drug diffusion and release, Marui et al., studied a pH‐responsive NP‐hydrogel composite material made of drug‐loaded PEG‐*b*‐(polylactic acid) polymeric NPs and an agarose/carbomer based hydrogel, which exhibited negative charge at physiological pH 7.4 and nearly neutral charge at acidic pH 5.^[^ ^161^ ^]^ Both NP diffusion out of the hydrogel and the diffusion of a model drug, Rhodamine B, loaded in the NPs embedded in the hydrogel were studied. By tuning the surface charge of the NPs via various surfactants, a fast release of negatively charged NPs from the hydrogel at pH 7.4 was observed, while no release of positively charged NPs was observed. Furthermore, neither positively nor negatively charged NPs were released from the hydrogel at pH 5, which indicates all the NPs were entrapped in hydrogel matrix via electrostatic interaction.

#### 5.2.3 Magnetic Field

Magnetic field‐controlled drug release offers a highly versatile and minimally invasive strategy for localized cancer therapy. This approach offers precise spatial and temporal control over drug delivery, enhances therapeutic targeting and tissue penetration,^[^ ^114^ ^]^ minimizes systemic toxicity, and enables noninvasive, on‐demand activation,^[^ ^60^ ^]^ making it an ideal platform for improving safety and outcomes in bioimaging and cancer therapy.^[^ ^234^ ^]^ Magnetic NPs, such as iron oxide NPs,^[^ ^235^ ^]^ are often embedded in hydrogel network, and the drug delivery can be controlled by applying on/off magnetic field to enable on‐demand drug release, Moreover, the hyperthermia effect of magnetic NPs under magnetic field provides a synergic therapeutic effect on cancer therapy^[^ ^62^ ^]^ and provides distinct advantages over passive drug release. For example, Sumitha et al., developed a NP‐hydrogel hybrid material composed of iron oxide NPs and chitosan to deliver DOX for cancer therapy.^[^ ^60^ ^]^ The diffusion of DOX from the hydrogel was accelerated by applying the magnetic field of 50 mT on hybrid material with 25% weight of iron oxide NPs that 30% of embedded DOX was released in 60 min of magnetic field application, while minimal DOX release was observed without magnetic field. Additionally, a stepwise increasing in cumulative DOX release was observed under 5 min on/off pulsatile magnetic field, which improved the DOX release from 1.2% to 31.5% in 60 min. While exposed to magnetic field, the different orientation of NPs in hydrogel network triggers the expansion and contraction of porous network, promotes the drug release due to fast diffusion.^[^ ^236^, ^237^ ^]^

#### 5.2.4 Redox Reaction

Reduction‐oxidation (Redox) reactions and ROS play a significant role in physiological and pathological processes, and can regulate the application of composite materials in tissue regeneration and wound healing.^[^ ^238^, ^239^ ^]^ ROS can be delivered as a therapeutic compound to mediate tissue regeneration,^[^ ^240^ ^]^ or can be utilized as a stimuli to break down the chemical linkages between drugs, NPs and hydrogels, such as the disulfide bond, and trigger on‐site release of drugs.^[^ ^108^ ^]^ For example, lonidamine complex was synthesized in the form of a dimer via a sulfur bond and then the drug complex was loaded into triphenylphosphine‐modified poly(L‐lactic acid) NPs via hydrophobic interaction. The drug loaded NPs were embedded in a DOX‐loaded HA hydrogel for combined chemo‐immune therapy. The sulfur bonds in the lonidamine dimer are responsive to GSH which accelerated the lonidamine release (90% release over 50 h) and achieved specific release in the GSH‐rich mitochondria organelle.^[^ ^57^ ^]^

### 5.3 Release via Hydrogel Degradation and Stimuli‐Responsive Hydrogels

Release of drugs or drug‐loaded NPs from a composite material can be controlled by the deformation of the hydrogel scaffold.^[^ ^241^, ^242^ ^]^ Deformation or degradation of the hydrogel scaffold can occur via simple passive swelling and hydrolysis of the hydrogel, or stimuli‐triggered degradation.^[^ ^206^, ^243^ ^]^

#### 5.3.1 Passive Degradation and Hydrolysis

Hydrogel composed of either natural polymers or synthetic polymers can exhibit passive degradation or hydrolysis in an aqueous environment.^[^ ^181^, ^244^ ^]^ The embedded NPs and drugs are released into the bulk media upon hydrogel breakdown. Therefore, controlling the hydrolysis and degradation of a hydrogel can influence the drug release rate whereby a fast degradation will lead to burst release^[^ ^245^ ^]^ and a very stable hydrogel can serve as a depot release system with prolonged release characteristics from days to months.^[^ ^246^ ^]^ Park et al., has developed a polyvinylpyrrolidone (PVP) microneedle drug delivery system with R837‐loaded Pluronic F127 NPs for cancer immunotherapy via transdermal administration. The PVP microneedle was designed to dissolve quickly and the drug loaded NPs released shortly after microneedles penetrated the skin and activated an immune response.^[^ ^101^ ^]^ Furthermore, Villalva et al., immobilized phytantriol cubosomes in a HA‐based hydrogel and no release of the lipid cubosomes was observed until a day before the hydrogel completely degraded.^[^ ^118^ ^]^ The hydrogel structure was enhanced by increasing the degree of HA crosslinking with adipic dihydrazide as the crosslinker. The hydrogel durability was improved at high crosslinking densities, leading to a slower degradation (completely intact for 168 h) than hydrogel with low crosslinking density (intact for 24 h).^[^ ^118^ ^]^

#### 5.3.2 Stimuli Triggered Hydrogel Degradation

Drug release from NP‐hydrogel hybrid materials can be controlled by the stimuli responsiveness of the hydrogel scaffold, Stimuli‐responsive hydrogels are designed to respond to physical, chemical, or biological stimuli, such as changes in temperature, light, electric field, magnetic field, ROS, pH, and enzyme.^[^ ^247^ ^]^ These hydrogels degrade, swell, or shrink in response to the external stimuli, thereby changing the effective mesh size and scaffold structure and controlling the release of the embedded drugs or drug‐loaded NPs.^[^ ^125^ ^]^

The hydrogel network can be degraded by the stimuli‐induced breakdown of the crosslinker. For example, mangiferin‐loaded polymeric NPs were embedded in a crosslinked hydrogel comprised of caffeic acid‐grafted *ε*‐polylysine and phenylboronic acid‐grafted oxidized dextran coloaded with diclofenac sodium (DS).^[^ ^125^ ^]^ The hydrogel was crosslinked by the Schiff base bonds and the boronic ester bonds, which are highly responsive to pH and ROS. The release data indicated that the release of DS and mangiferin were increased to 84.5% and 71.1% over 7 days at pH 5 and 1 mm hydrogen peroxide, respectively, due to the hydrolysis of the crosslinker and the degradation of the hydrogel.^[^ ^125^ ^]^ Similarly, carboxymethyl chitosan hydrogel was crosslinked with miRNA inhibitor‐loaded tannic acid NPs via boronic ester bonds. The crosslinking ester bonds broke down at acidic pH 5 and in the presence of hydrogen peroxide, upon which the hydrogel matrix fell apart and the embedded miRNA drug was fast released up to 85% over the first week.^[^ ^102^ ^]^

The hydrogel network can also be degraded by the breakdown of the polymer chains due to the stimuli responsiveness of the polymers.^[^ ^248^ ^]^ For example, chitosan‐based hydrogel embedded with silica NPs and ciprofloxacin was developed by Zare et al.,^[^ ^116^ ^]^ and the release of ciprofloxacin was accelerated from 86% at pH 7.4 over 86 h to 97% at pH 4.2 over 48 h. It is because at low pH, protonation of the amino groups on the chitosan polymer leads to electrostatic repulsion between each polymer chain, promoting polymer chain expansion and facilitating water diffusion into the gel. The enlarged pores in the hydrogel network improved the diffusion and release of the encapsulated drug and drug‐loaded NPs.^[^ ^117^ ^]^

Hydrogels can also be digested by enzymes,^[^ ^57^ ^]^ and this mechanism can take advantage of the presence of specific enzymes at a biological interface. For example, Cisplatin‐loaded trilaurin LNPs were encapsulated in a dextran‐based hydrogel network,^[^ ^56^ ^]^ which can be degraded with the enzyme dextranase as a trigger at the colon enabling a specific targeted anticancer drug release at the colon for tumor immunotherapy and chemotherapy.

Drug release from hydrogels of temperature‐sensitive polymers can be controlled by the temperature change via the heat generated by the NPs with photothermal effects.^[^ ^60^, ^112^, ^113^, ^133^ ^]^ Temperature‐sensitive hydrogels, including poly(*N*‐isopropylacrylamide) (PNIPAm) and gelatin, experience a coil‐globule phase change with temperature changes.^[^ ^249^ ^]^ PNIPAm is one of the most well‐studied temperature‐sensitive hydrogels and it is known that the hydrogel network shrinks to a globule state at temperatures above the lower critical solution temperature (LCST) and swells to a coil state at temperatures below the LCST.^[^ ^250^, ^251^ ^]^ The swell/shrink behavior occur because of the formation and dissociation of hydrogen bonds between the amide groups on PNIPAm and the free water molecules. Chen et al., developed a thermo‐responsive hybrid material by incorporating cellulose nanocrystals decorated with Fe_3_O_4_ nanoparticles into PNIPAm hydrogel.^[^ ^138^ ^]^ Then, free drug vancomycin was loaded to the preformed hydrogel by passive adsorption. The release of vancomycin was accelerated from 52% (NIR off) to 74% (NIR on), and an on/off activation of vancomycin release was also observed.^[^ ^138^ ^]^ Similarly, Lv et al., developed a thermo‐ and magnetic‐sensitive drug delivery system by coating acetylsalicylic acid (AA) intercalated magnetic ZnAl layered double hydroxides (LDH) core with a layer of AA‐PNIPAm micelle and a layer of lactic acid (LA) intercalated magnetic ZnAl‐LDH via the layer‐by‐layer deposition technique to deliver the anti‐inflammatory molecule AA.^[^ ^252^ ^]^ The release of AA at lower temperature (25 °C) was significantly higher than at higher temperature (40 °C). Notably, due to differences in loading strategies, the temperature‐responsive release profiles can exhibit significant variations. In the previous study, vancomycin was loaded to the preformed PNIPAm hydrogel via passive adsorption,^[^ ^138^ ^]^ thus it can be easily squeezed out by a shrinking hydrogel. As a result, increasing temperature accelerated the drug release. On the other hand, when AA was loaded in hydrogel via a layer‐by‐layer deposition technique,^[^ ^252^ ^]^ shrinking PNIPAm decreased the interlayer space and hydrogel pores, collapsed the interlayer cavity, and led to a delayed and limited drug release at high temperature.

## 6 Conclusion and Perspectives

The integration of NPs into hydrogel matrices has demonstrated significant potential for controlled and stimuli‐responsive drug delivery applications.^[^ ^253^ ^]^ This hybrid system combines the structural advantages of hydrogels with the functional properties of NPs and shows an outstanding positive impact on drug delivery and controlled drug release over NP or hydrogel system alone. The review highlights the critical role of molecular interactions (e.g., electrostatic, hydrophobic, and hydrogen and covalent bonding) in governing drug/NP embedment and diverse stimuli‐responsive mechanisms of releasing therapeutic payloads in a controlled manner. Incorporating NPs in hybrid materials enables the encapsulation of hydrophobic drug, such as DOX, and sensitive molecules, such as mRNA and DNA, enhances the overall mechanical strength, introduces novel functionality and promotes on‐demand drug release with external stimuli. On the other hand, hydrogel coating protects the NPs, promotes on‐target NP and drug retention, and increases biocompatibility. While advancements in hybrid systems have enabled precise spatiotemporal control over drug delivery, some knowledge gaps and translational challenges still remain to be addressed with ongoing research.

Interactions between the embedded NPs and the hydrogel network are generally poorly characterized and understood, and research tends to focus on tracking the release of the loaded drugs instead of the release behavior of NPs. However, the molecular interactions between NPs and hydrogels are an essential aspect in materials design and selection as reviewed.^[^ ^254^ ^]^ This lack of focus on NP release could be due to technological difficulties of tracking NPs in various environments compared to drug molecules. Fluorescence monitoring is commonly used for dye‐labeled NPs.^[^ ^255^, ^256^ ^]^ However, photobleaching, degradation, quenching, and leakage of the dye from NPs may all lead to an inaccuracy of the measurement.^[^ ^257^, ^258^, ^259^ ^]^ As a result, technological innovation in tracing NPs within developed hydrogel networks could provide benefit, and future research should focus not only on the release of bioactives but also on NP release behavior to produce a more thorough understanding of the mechanism of NP loading and release.

Another promising research direction involves the incorporation of drug‐loaded NPs in microgels or nanogels. While extensive research has been conducted on the incorporation of NPs into conventional hydrogel systems, combining NPs with microgels and nanogels presents a new avenue of research that remains largely unexplored. Microgels and nanogels have recently gained attention as an alternative to bulk hydrogels in various applications.^[^ ^71^, ^260^, ^261^ ^]^ Microgels are characterized by their excellent tunability, microporosity, and designed heterogeneity, which stem from their modular nature^[^ ^262^, ^263^, ^264^ ^]^ and nanogels have specific high drug loading capacity,^[^ ^265^ ^]^ and targeted delivery capability. The small size and customizable properties of microgels and nanogels enable precise control over the release kinetics of the loaded drugs. Additionally, multilayer hydrogels can be designed to degrade at different rates, allowing for a controlled, sequential, and sustained release of drugs over time^[^ ^266^, ^267^, ^268^ ^]^ and incorporating NPs may further refine this release profile. Furthermore, using microgels to fabricate granular microporous hydrogels has attracted attention in improving the performance of hydrogel scaffolds due to their larger pore size.^[^ ^64^ ^]^ Integration of NPs within these new types of hydrogels may hold potential for the development of more advanced drug delivery systems.

The rising clinical use and potential of mRNA‐LNP technology in biomedical applications is an exciting area that should also be explored by combining them with a hydrogel depot system. As this review presents, there has been very limited studies integrating the new type of mRNA‐loaded ionizable LNPs into a hydrogel system. Despite the success of the clinically used SARS‐CoV‐2 mRNA vaccines,^[^ ^269^ ^]^ key challenges remain in optimizing mRNA delivery, particularly in improving stability for easier storage and transport and enhancing mRNA delivery efficiency.^[^ ^270^ ^]^ Meany et al., reported that the mRNA‐loaded LNP vaccines can be protected from RNases and other enzymes when embedded within a hydrogel network which restricts enzyme diffusion, and thus obtaining sustained release and expression of mRNA and improving immune response.^[^ ^270^ ^]^

In conclusion, NP‐hydrogel hybrid materials leverage the advantages of both hydrogels and NPs. The ability of these composite materials to respond to external stimuli enables control over drug release kinetics and provide an ability to address critical challenges in therapeutic agent delivery. However, despite considerable progress, challenges remain in optimizing drug‐NP‐hydrogel interactions, ensuring precise and accurate tracking of the release of the therapeutic agents and/or NPs, enabling on‐demand drug release and improving biocompatibility and stability. Continued research efforts focusing on progressing mechanistic understandings and material engineering will be essential for advancing these systems toward clinical applications. The development of next‐generation NP‐hydrogel composites holds promise for significantly enhancing drug delivery efficiency, minimizing systemic side effects, and broadening their application across various biomedical fields.

## Conflict of Interest

The authors declare no conflict of interest.
